# Joint contributions of the gut microbiota and host genetics to feed efficiency in chickens

**DOI:** 10.1186/s40168-021-01040-x

**Published:** 2021-06-01

**Authors:** Chaoliang Wen, Wei Yan, Chunning Mai, Zhongyi Duan, Jiangxia Zheng, Congjiao Sun, Ning Yang

**Affiliations:** 1 National Engineering Laboratory for Animal Breeding and Key Laboratory of Animal Genetics, Breeding and Reproduction, Ministry of Agriculture and Rural Affairs, China Agricultural University, Beijing, 100193 China; 2 National Animal Husbandry Service, Beijing, 100125 China

**Keywords:** Chicken, Feed efficiency, Genetic variations, Gut microbiota, Spatial heterogeneity

## Abstract

**Background:**

Feed contributes most to livestock production costs. Improving feed efficiency is crucial to increase profitability and sustainability for animal production. Host genetics and the gut microbiota can both influence the host phenotype. However, the association between the gut microbiota and host genetics and their joint contribution to feed efficiency in chickens is largely unclear.

**Results:**

Here, we examined microbial data from the duodenum, jejunum, ileum, cecum, and feces in 206 chickens and their host genotypes and confirmed that the microbial phenotypes and co-occurrence networks exhibited dramatic spatial heterogeneity along the digestive tract. The correlations between host genetic kinship and gut microbial similarities within different sampling sites were weak, with coefficients ranging from − 0.07 to 0.08. However, microbial genome-wide analysis revealed that genetic markers near or inside the genes *MTHFD1L* and *LARGE1* were associated with the abundances of cecal *Megasphaera* and *Parabacteroides*, respectively. The effect of host genetics on residual feed intake (RFI) was 39%. We further identified three independent genetic variations that were related to feed efficiency and had a modest effect on the gut microbiota. The contributions of the gut microbiota from the different parts of the intestinal tract on RFI were distinct. The cecal microbiota accounted for 28% of the RFI variance, a value higher than that explained by the duodenal, jejunal, ileal, and fecal microbiota. Additionally, six bacteria exhibited significant associations with RFI. Specifically, lower abundances of duodenal *Akkermansia muciniphila* and cecal *Parabacteroides* and higher abundances of cecal *Lactobacillus*, *Corynebacterium*, *Coprobacillus*, and *Slackia* were related to better feed efficiency.

**Conclusions:**

Our findings solidified the notion that both host genetics and the gut microbiota, especially the cecal microbiota, can drive the variation in feed efficiency. Although host genetics has a limited effect on the entire microbial community, a small fraction of gut microorganisms tends to interact with host genes, jointly contributing to feed efficiency. Therefore, the gut microbiota and host genetic variations can be simultaneously targeted by favoring more-efficient taxa and selective breeding to improve feed efficiency in chickens.

**Video abstract**

**Supplementary Information:**

The online version contains supplementary material available at 10.1186/s40168-021-01040-x.

## Background

Demand for animal-source foods is increasing because of population growth, rising household income and urbanization. Chicken meat is a white meat, distinguished from other meats such as pork, beef, and mutton by its lower content of undesirable saturated fat [1]. The global consumption of chicken meat has shown the fastest growth trend in recent decades because of its consistently high-quality and relatively low price [2]. More than 72 billion broiler chickens were produced and chicken had become the largest meat producer worldwide in 2019 (FAOSTAT). However, this production level has mainly been achieved using high-quality feed ingredients, such as maize, soybean, and wheat [3]. Feed accounts for nearly 70% of the total variable costs in modern chicken production. In recent years, these ingredients have generally become more expensive because of a combination of increased demand from human nutrition [4], biofuel production [5], and shortages due to crop failures in parts of the world [6]. With continuing reliance on the same feed ingredients, which compete with human consumption and biofuel needs, the cost of chicken production will also increase. Hence, to meet the increasing demand for chicken meat, the efficiency of converting feed into edible products should be improved.

Feed efficiency can be evaluated by different measures. Among them, residual feed intake (RFI) is independent of growth traits, making it the most suitable indicator for feed efficiency [7–9]. Chickens that have low RFI values are more efficient than those with high values. As a complex feed efficiency trait, RFI is influenced by various factors. Numerous studies have reported that RFI shows moderate heritability (0.26~0.45) in chickens [9–12], implying that host genetics play an important role in regulating feed utilization. Zuidhof et al. [13] showed that the feed efficiency of commercial breeds improved by 50% over the last 50 years due to quantitative genetic selection pressures. Another factor that could markedly affect animal feed efficiency is the gut-residing microbiota, which is a functional entity that influences host metabolism [14]. Growing evidence has confirmed that the relative proportions of digestion and energy harvesting from feed are affected by the gut microbial activity and composition [15–17]. In particular, the saccharolytic and anaerobic microbiota can degrade host-indigestible carbohydrates, such as cellulose and resistant polysaccharides, into monomeric or dimeric sugars and subsequently ferment them into short-chain fatty acids (SCFAs) [18, 19]. Most of these metabolites are absorbed by the host and contribute to its energy [19]. Hence, identifying a more energy-efficient microbiota is necessary for the development of effective strategies to improve feed utilization and preserve additional edible resources for humans.

Indeed, as described by previous observations, the gut microbiota is closely related to feed efficiency in chickens [20–22]. However, the association between host genetics and gut microbiota in feed efficiency is poorly understood. A few recent studies have suggested that divergent genetic selection for digestive efficiency has led to differences in cecal microbial ecosystems between chickens with high and low feed efficiencies [23, 24]. Likewise, several studies in cattle have identified some rumen microbiota that are heritable and associated with feed efficiency [25, 26]. However, it is largely unknown whether host genetics affect feed utilization through their ability to promote a stable microbial community in the gut or whether the two interact to influence feed efficiency. If the relative abundance of the RFI-related microbiota across individuals is attributable to host genetic effects, detecting host genetic markers as biomarkers for manipulating gut microbial composition may be possible. Evidence from genome-wide association studies (GWAS) has identified several host genetic variations that affect the gut microbiota [25, 27–30]. For example, 19 single-nucleotide polymorphisms (SNPs) were associated with 14 rumen microbial taxa in cattle [25]. Bergamaschi et al. [31] observed that several SNPs were significantly associated with gut taxa at the three time points during the growth trial in pigs. Org et al. [27] reported that seven genome-wide significant loci were associated with genera abundance in mice. Substantial studies in the human population have demonstrated that the abundance of the genus *Bifidobacterium* is strongly related to loci within the lactase gene region [28–30]. However, potential host genotypes relevant to the gut microbiota in chickens have not been well characterized. Recently, Psifidi et al. [32] reported significant associations between host genetic variation and alpha diversity and beta diversity axes, indicating the possibility of host genetic variation shaping gut microbial composition in chickens.

**Fig. 1:**
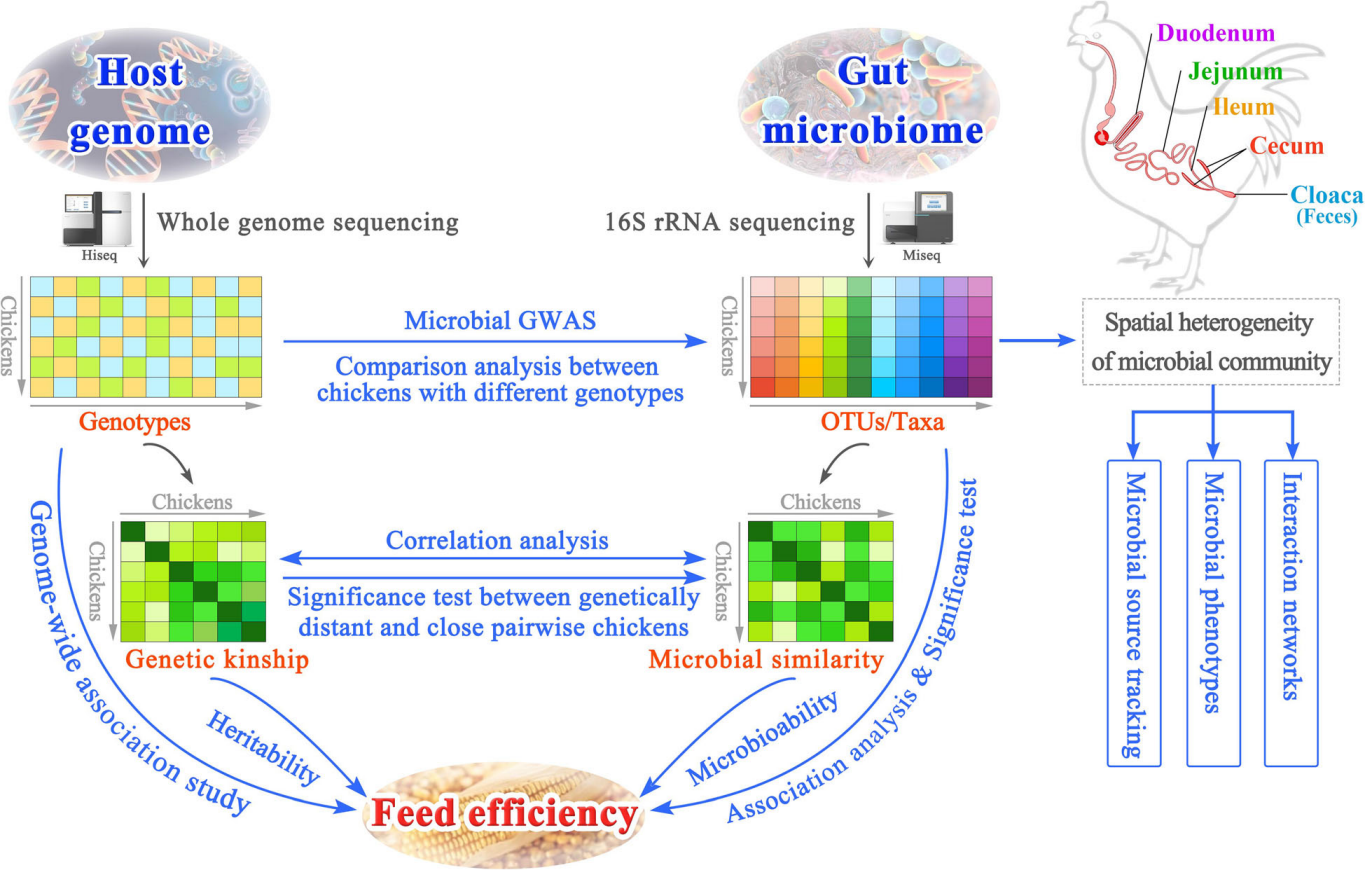
Analysis workflow of the present study

Fecal specimens are frequently used as proxies for the gut microbiota, while the functional heterogeneity of each digestive tract segment gives rise to regional differences in gut microbial populations [33, 34]. In poultry, nutrient digestion and absorption primarily occur in the small intestine (including the duodenum, jejunum, and ileum), and the cecum is a major site of the fermentation of dietary materials. Therefore, evaluating the contributions of host genetics and gut microbiota from diverse segments to feed efficiency will aid in understanding the underlying biological variation in feed efficiency and consequentially help in the design of sustainable approaches to improve feed efficiency in chickens. To achieve this goal, we used host genetic data and the microbial taxa in four gut segments (duodenum, jejunum, ileum, and cecum) and feces of 206 meat-type chickens to clarify the spatial relationship between the microbiota and its hosts, assess their joint effect on feed efficiency, and further identify the specific genetic variation and microbiota that are significantly associated with feed efficiency. The overall flow of the analyses is shown in Fig. 1.

## Methods

### Animals and sample collection

A total of 206 male chickens from a purebred line were used in the present study. This strain belonged to the yellow-feather dwarf broiler breed, which is an important sire line for Guangdong Wen’s Nanfang Poultry Breeding, Co., Ltd. (Xinxing, China). All the chickens were hatched on the same day and housed on floor pens with fresh wood sawdust as litter. Water and corn-soybean-based diets (the ingredients of the diets are included in Additional file 1: Table S1) were provided ad libitum. Each chicken was properly identified by their unique electronic chip. The feed intake was individually recorded using the automatic feeder that registered visits to feeders by identifiable chickens during the fast-growing period from 56 to 76 days of age. The body weight of each chicken at 56 and 76 days of age was measured using an electronic scale. RFI was calculated based on the average daily feed intake (ADFI), average daily gain (ADG), and metabolic mid-weight as described by Yan et al. [35]. Normality for all the traits was checked using the Shapiro-Wilk test in the R program (ver 4.0.2). The descriptive statistics of these phenotypes are summarized in Additional file 2: Table S2. The correlation coefficient between RFI and ADFI was 0.60, and the correlation of RFI with ADG was negligible (Additional file 3: Figure S1).

At the age of 78 days, the whole blood of each bird was collected from the wing vein using a syringe, and the fecal specimen was gathered from the cloaca by squeezing the abdomen (Additional file 4: Figure S2). Then, all the birds were euthanized by cervical dislocation and dissected. The duodenal, jejunal, ileal, and cecal contents (including chyme and mucosa) were collected immediately. All the samples were snap frozen in liquid nitrogen and then stored long-term at − 80 °C until further processing.

### High-throughput sequencing and sequence processing

The host DNA and gut microbial DNA were isolated using the Tiangen DNA Extraction Kit (Tiangen Biotech, Beijing, China) and QIAamp DNA Stool Mini Kit (QIAGEN, Hilden, Germany), respectively. One blood sample and four fecal samples were excluded because of DNA extraction failure. Finally, 205 host DNA samples and 1026 microbial DNA samples were used for sequencing. The details of the whole-genome sequencing of the host and 16S rRNA sequencing of microbiota have been previously described [33]. Briefly, the host DNA library was paired-end sequenced with 150-bp read lengths and a 10-fold depth. The V4 region of the 16S rRNA gene was amplified using the universal primers 520F and 802R and then sequenced with 2 × 300-bp read lengths.

To avoid reads with artificial bias, quality control was conducted using FastQC (ver 11.7). The clean reads of the host were mapped on the reference genome (Galgal5) using BWA mem (ver 0.7.15) [36]. We further removed the duplicates using the Picard toolkit (ver 1.119, https://broadinstitute.github.io/picard/). Subsequently, The Genome Analysis Toolkit (GATK, ver 3.7) [37] was used for SNP calling, following GATK best practices, in which realignment and recalibration were included. SNP calling and genotyping were performed using the HaplotypeCaller module embedded in GATK. To ensure accuracy in variant calling, a minimum quality score for both base quality and mapping quality was set to 20. The average genome coverage was 95.25%, which allowed us to call variants with high coverage. Stringent filtering criteria were applied to the concordant part of the biallelic SNPs using the VariantFiltration module. Because alleles at lower frequencies are less informative for association analysis, we retained only SNPs with minor allele frequencies (MAFs) above 5% and kept only SNPs that occurred in more than 95% of individuals. The final set included 9,335,193 SNPs (87.82% of the SNPs were found in the SNP database), which were used in the downstream analysis. The principal component analysis on these SNPs is displayed in Additional file 5: Figure S3. The chicken gene set was downloaded from the Ensembl database (release 91), and gene-based annotation of valid SNPs was conducted using ANNOVAR [38] (https://annovar.openbioinformatics.org/). In total, 4,643,107 (49.74%) and 4,427,712 (47.73%) SNPs were mapped to intergenic and genic regions, respectively (Additional file 6: Table S3).

The paired-end reads of the microbiota were processed and clustered into operational taxonomic units (OTUs) using the QIIME (ver 1.8.0) pipeline [39]. In brief, raw reads with exact matches to the barcodes were assigned to the respective samples and identified as valid sequences. The low-quality reads were removed based on the following criteria: (1) read lengths < 150 bp; (2) contained ambiguous bases; (3) contained mononucleotide repeats > 8 bp; (4) average quality score < 20. The high-quality paired-end reads with an overlap > 10 bp and without any mismatch were assembled using FLASH [40]. Taxonomies were assigned to OTUs at 97% sequence identity using an open-reference OTU picking protocol with the SILVA database [41]. Only OTUs with an average relative abundance greater than 0.0001% and detected in at least two samples were included in the downstream analysis. Additional details about the host and microbial sequence processes are found in Additional file 7: Text S1.

### Characterizing the spatial heterogeneity of the gut microbial community

Multiple comparisons of the observed OTUs among the five sample types (duodenum, jejunum, ileum, cecum, and feces) were conducted by one-way analysis of variance (ANOVA) followed by Tukey’s HSD test using the multcomp package in the R program. The difference was considered statistically significant if the adjusted *P* value was less than 0.05. The overall dissimilarities among the diverse gut locations were evaluated by nonmetric multidimensional scaling (NMDS) according to Bray-Curtis dissimilarity matrices at the family taxonomic level. To determine the potential origin of the microbiota found in the gut contents, we used the microbial source-tracking method FEAST [42] (https://github.com/cozygene/FEAST) in the R program. FEAST is a highly efficient expectation-maximization-based method that estimates the fraction of a microbial community contributed by a potential source environment. Each sampling site was identified as a sink, starting with the ileum, and all anterior segments were treated as sources.

To compare the oxygen tolerances and biofilm formation capabilities of microbial communities in the four gut segments and feces, the abundances of aerobic, anaerobic, facultatively anaerobic, and biofilm-forming bacteria were predicted using BugBase [43]. The significances of the comparisons were determined using the pairwise Wilcoxon rank-sum test. The correlations between the detected taxa in a specific sample type were inferred using the corr.test function in the psych package and *P* values were adjusted using the Benjamini–Hochberg (BH) method. To avoid potential bias in the co-occurrence calculations caused by zero inflation, the taxa that were present in less than 95% of the samples of a specific sample type were eliminated from the co-occurrence network analysis. The relative abundance of each microbial taxon was log_10_-transformed and subsequently used to calculate the correlation coefficients. The correlation patterns were further filtered to select only Spearman’s and Pearson’s correlations with coefficients < − 0.3 or > 0.3 and adjusted *P* values < 0.05. Interaction networks were then constructed using Cytoscape (ver 3.7.2) [44].

### Investigation of the association between host genetics and the gut microbiota

The host genetic kinship was estimated based on the host genotyping data as previously described [33]. We and others have previously estimated the relationship between host genetic kinship and microbial similarity based on all sample data sets [30, 33, 45]; however, most of the host genetic relatedness between individuals was weak, which may affect the estimated reliability. Therefore, in the present study, pairs of chickens with an estimated genetic kinship ≤ 0.05 and ≥ 0.10 were considered genetically more distant and close relatives, respectively [30, 46]. In total, 20,910 pairs of individuals (${C}_{205}^2$) were included in our study, and we randomly sampled 500 pairs of more distant relatives and 500 pairs of closer relatives from this dataset using the dplyr package. Subsequently, the correlations between the host genetic kinship and Bray-Curtis similarity of these pairs of chickens were calculated. ANOVA was used to determine the differences in microbial diversity between more distant and close relatives. We repeated this process 10,000 times and summarized all the estimated correlation coefficients via their density distribution using a customized R script. The lowest and highest 2.5% of values were removed to generate a 95% confidence interval for the correlation coefficients for a given sample type.

In addition to evaluating the correlation between host genetics and gut microbial similarity, heritability estimates have been used as efficient measures for determining the influence of genetics on a specific taxon. In our previous study, we have identified 58 heritable microbial genera [33]. Thus, we further performed GWAS analysis to detect significant host genetic markers affecting the microbial genera using the following linear mixed model in GEMMA (ver 0.98.1) [47]:

1 $$ \mathbf{y}=\mathbf{Q}\boldsymbol{\upalpha } +\mathbf{X}\beta +\mathbf{g}+\mathbf{e} $$

where **y** is a vector of corrected phenotypes (the abundance or presence/absence of heritable genera) as previously described [33]; **Q** is a design matrix of covariates, including the top five host genetic principal components calculated as previously described [33]; **α** is a vector of effects for the covariates (including the intercept); **X** is a vector of allele counts (0, 1, 2); and *β* is the SNP effect. **g** is a vector of polygenic effects that follows the normal distribution *N*(0, **G**${\sigma}_g^2$), where **G** is the genetic kinship matrix calculated from genome-wide marker information and ${\sigma}_g^2$ is the polygenic additive variance. **e** is a vector of residual errors. The *P* values of the SNP effects were calculated using the likelihood ratio test. After the Bonferroni correction, the genome-wide significance threshold was set at 5.36 × 10^−9^ (0.05/9,335,193). However, the Bonferroni correction is very strict. We calculated the effective number of independent tests using simpleM [48], and 180,042 independent tests were suggested. Thus, the suggestive genome-wide significance was set at 2.78 × 10^−7^ (0.05/180,042). Given that the new reference genome (galGal6) is available, the coordinates of these significant or suggestive significant SNPs were converted from galGal5 to galGal6 with the liftOver tool (http://www.genome.ucsc.edu/cgi-bin/hgLiftOver).

Notably, a genomic region containing a cluster of neighboring SNPs in strong linkage disequilibrium (LD) is usually associated with a phenotype for a high-density array. To demarcate independent association signals across the putative regions, we performed LD analysis to further characterize causative SNPs associated with target traits using PLINK (ver 1.9) [49]. Because the average LD level in a 5-kb interval was 0.17~0.24 (Additional file 8: Figure S4), pairs of SNPs with *r*^2^ greater than 0.2 were regarded as highly linked.

### Evaluating effects of host genetics and the gut microbiota on feed efficiency

To estimate the contributions of host genetics to feed efficiency, the variance in RFI explained by all the genotyped SNPs was estimated by restricted maximum likelihood analysis of the following model implemented in GCTA (ver 1.91.1) [50]:

2 $$ \mathbf{y}=\mathbf{Q}\boldsymbol{\upalpha } +\mathbf{g}+\mathbf{e} $$

where the model parameters were as described in model (1) except for **y**, which is a vector of RFI phenotypes. Genomic heritability is defined as *h*^2^ = ${\sigma}_g^2$/${\sigma}_p^2$, where ${\sigma}_p^2$ is the phenotypic variance. Furthermore, model (1) was fitted to detect host genetic variations related to feed efficiency. After detecting SNPs that are associated with RFI, we further investigated the effects of host genotypes on the gut microbiota. We extracted these RFI-related SNPs and explored the differences in each microbial abundance among chickens with different genotypes using the Wilcoxon rank-sum test. The *P* values were adjusted using the BH method with the p.adjust function in R. The difference was considered significant at adjusted *P* value < 0.05.

To assess the proportion of variation in feed efficiency due to the microbiota from diverse sampling sites, a microbial relationship matrix was constructed based on the OTU abundance as previously described [33]. The following model was fitted to estimate the variance explained by the microbial community:

3 $$ \mathbf{y}=\mathbf{Q}\boldsymbol{\upalpha } +\mathbf{m}+\mathbf{e} $$

where **y** is a vector of RFI phenotypes, **Q** is a design matrix of covariates, and **α** is a vector of effects for the covariates. The covariates included the top five host genetic principal components and first three principal components of significant and suggestive significant SNPs associated with RFI. **m** is a vector of the random effects of the microbiota in the specific sampling site that follows the normal distribution *N*(0, **M**${\sigma}_m^2$), where **M** is the microbial relationship matrix and ${\sigma}_m^2$ is the microbial variance. **e** is a vector of residual errors. The proportion of the total variance explained by the gut microbiota is called the microbiability [33, 51, 52] and is defined as *m*^2^ = ${\sigma}_m^2$/${\sigma}_p^2$, where ${\sigma}_m^2$ is the microbial variance.

### Identification of the specific microbiota associated with feed efficiency

Since taxa at lower detection rates are less informative for association analysis, we retained only taxa that presented in a specific sample type in more than 30% of samples. The associations between qualified taxa and RFI were analyzed using a two-part model with a customized R script as described by Fu et al. [53]. This model accounts for both binary (present and absent) and quantitative features and is described as follows:

4 $$ y=\left\{\begin{array}{c}\ {\beta}_1\mathrm{b}+\mathrm{e}\ \\ {}{\beta}_2\mathrm{q}+\mathrm{e}\end{array}\right. $$

where *y* is the RFI value, *b* is a binary feature of a specific microorganism and coded as 0 for absent or 1 for present for each sample, and *q* is the log_10_-transformed abundance of a specific microorganism. *β*_1_ and *β*_2_ are the regression coefficients for the binary and quantitative models, respectively, and *e* is the intercept. The second part of the quantitative analysis was only for the samples in which the specific microorganism was present. The details of the two-part model are illustrated in Additional file 9: Figure S5. *P* values were obtained from the two-part model association analysis and adjusted by the BH method. If the adjusted *P* value from the binary model was less than 0.05, the presence or absence of microorganisms could influence feed efficiency. If the adjusted *P* value from the quantitative model was less than 0.05, feed efficiency was associated with the relative abundances of the microorganisms.

To detect specific microorganisms that significantly influenced feed efficiency, ANOVA was used to test the difference in RFI between chickens with the highest (*N* = 40) and lowest (*N* = 40) abundances of specific microorganisms. Additionally, the Wilcoxon rank-sum test was performed to determine the relative abundance of each taxon between the highest (*N* = 40) and lowest (*N* = 40) RFI-ranked chickens. A microorganism was considered significant if the adjusted *P* values from the two-part model association analysis, ANOVA and Wilcoxon rank-sum test were all less than 0.05. Furthermore, we explored the spatial distribution of the RFI-related microbiota among the four gut segments and feces. Spearman’s and Pearson’s correlations among the taxa were calculated using the psych package in R, and *P* values were adjusted using the BH method. Correlations were considered significant if the adjusted *P* value was < 0.05.

## Results

### Spatial heterogeneity of the gut microbial community

**Fig. 2:**
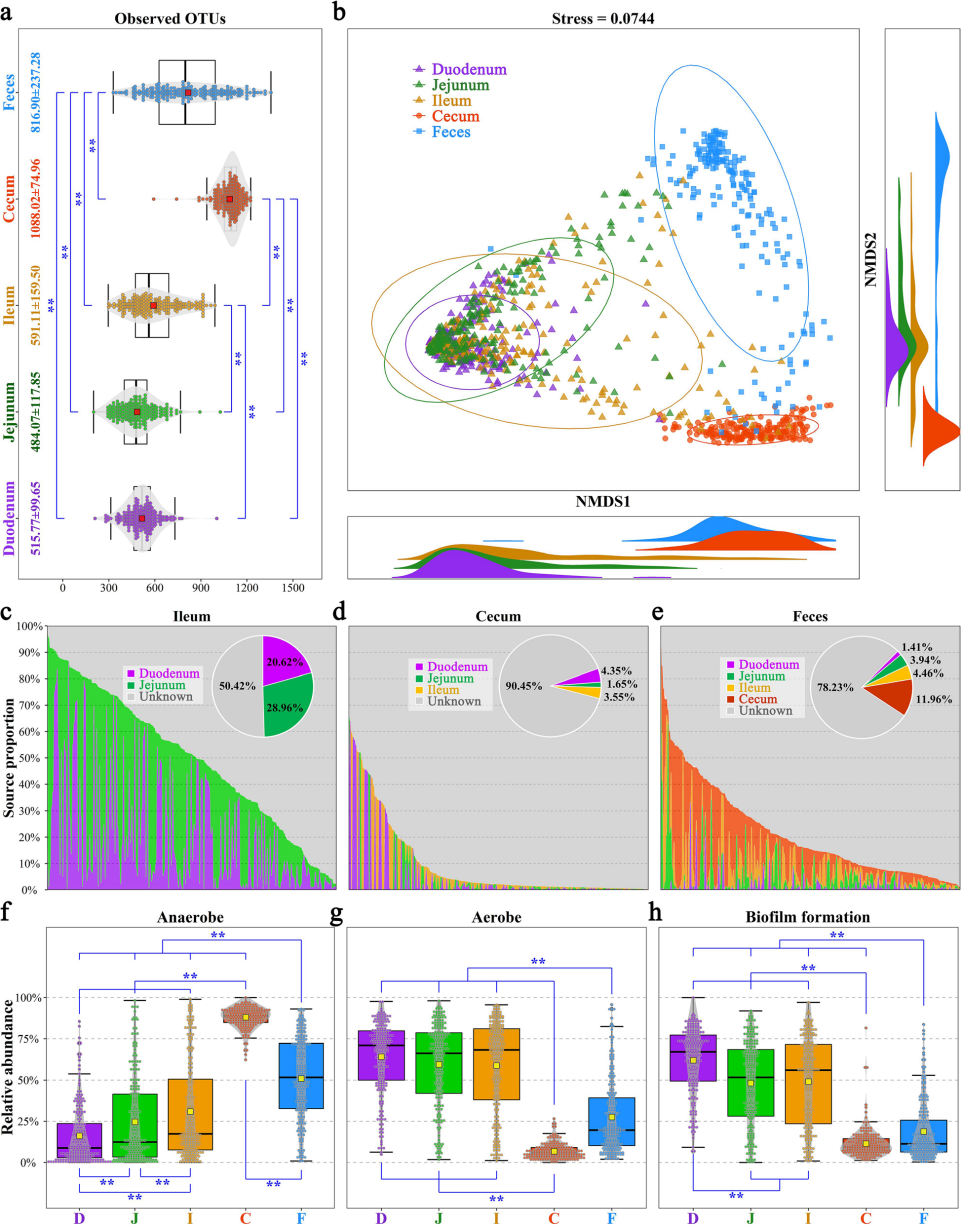
Spatial heterogeneity of the gut microbial community. **a** Comparison of the observed OTUs among the five sample types. Each point represents a sample. The center red point indicates the mean value in the corresponding sample type and the data are expressed as means ± SD. ** indicates an adjusted *P* value < 0.01. **b** Nonmetric multidimensional scaling (NMDS) at the family taxonomic level. Microbial source tracking: **c** ileum, **d** cecum, and **e** feces. Comparison of the relative abundance of **f** anaerobe, **g** aerobe, and **h** biofilm formation. D, J, I, C, and F represent the duodenum, jejunum, ileum, cecum, and feces, respectively. The center yellow point indicates the mean value in the corresponding sample type

The digestive tract contains several distinct habitats that select for the heterogeneous spatial organization of the resident microbiota. The observed OTUs differed significantly among the five sample types (except for the duodenum vs. ileum; Fig. 2a). Additionally, the gut microbial community clearly differed among the small intestine (duodenum, jejunum, and ileum), cecum, and feces because those groups clustered separately in an NMDS plot (Fig. 2b). The degree of dispersion for samples incrementally increased in each subsequent section of the small intestine: duodenum < jejunum < ileum. Microbial source tracking revealed that the ileal microbiota was predominantly sourced from the jejunal microbiota (28.96%), followed by the duodenum (20.62%); however, 50.42% of the source was still unknown (Fig. 2c). The cecal and fecal microbial communities showed no clear microbial sourcing from anterior gut segments (Fig. 2d, e), with unknown sources accounting for 90.45% and 78.23% of the totals, respectively, indicating a unique microbial community within cecum and feces.

We inferred the microbial phenotypes using BugBase and observed that oxygen tolerance differed significantly among the five sampling sites. Anaerobes were more abundant in the cecum and feces, with total abundances of 87.98% and 51.64% (Fig. 2f), respectively. By contrast, the three parts of the small intestine were dominated by aerobes, accounting for approximately 60% of the total abundance (Fig. 2g). Despite no differences in the abundance of aerobes among the three parts of the small intestine, anaerobes significantly increased from the duodenum to the ileum (from 16.11 to 30.88%). The relative abundances of facultative anaerobes in the five sampling sites were less than 15% (Additional file 10: Figure S6). In addition, the highest biofilm formation ability was observed in the duodenal microbiota, followed by the jejunal microbiota and ileal microbiota (Fig. 2h). However, biofilm formation was significantly reduced in cecal and fecal samples than in the small intestine.

**Fig. 3:**
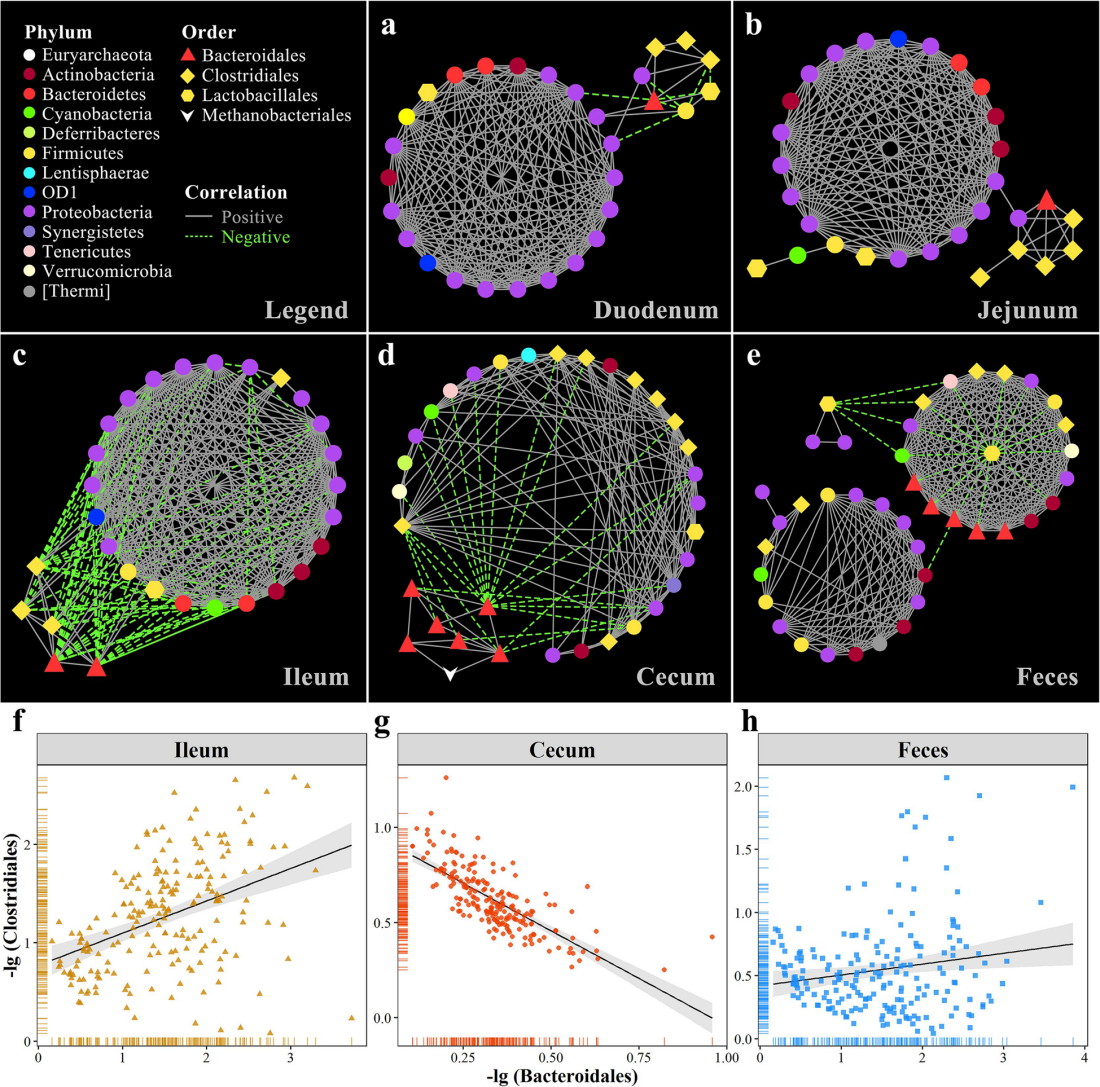
Gut microbial interaction network. Gut microbial co-occurrence network in the **a** duodenum, **b** jejunum, **c** ileum, **d** cecum, and **e** feces. Only correlations with coefficients < − 0.3 or > 0.3, and adjusted *P* values < 0.05 are displayed. The color and shape of nodes represent the phylum and order, respectively. The solid and dashed lines indicate positive and negative correlations, respectively. Relationship between the orders Bacteroidales and Clostridiales in the **f** ileum, **g** cecum, and **h** feces

Biofilms are not just bacterial slime layers but coordinated functional communities. Therefore, a co-occurrence network of core families in the five sampling sites was constructed to explore the spatial changes in microbial interactions. Positive correlations were observed in the core families of both the duodenum and jejunum (Fig. 3a, b and Additional files 11-12: Tables S4-S5). In the ileum, microbial families belonging to the orders of Bacteroidales and Clostridiales, which comprise anaerobes, competitively inhibited a cluster of bacteria, with significant and negative correlations with other families (Fig. 3c and Additional file 13: Table S6). However, microbial families in the cecum belonging to Bacteroidales were negatively correlated with most families belonging to Clostridiales (Fig. 3d and Additional file 14: Table S7). Additionally, the fecal microbial community showed two relatively independent and stable clusters (Fig. 3e and Additional file 15: Table S8). We further explored the interaction between Bacteroidales and Clostridiales and observed a moderately positive correlation in the ileum (*r* = 0.44, Fig. 3f), while a strong negative correlation was present in the cecum (*r* = − 0.78, Fig. 3g) and there was no significant correlation with each other in the feces (Fig. 3h). These results revealed that the clustering of the microbial co-occurrence network varied with the gut location.

### Association between host genetics and the gut microbiota

**Fig. 4:**
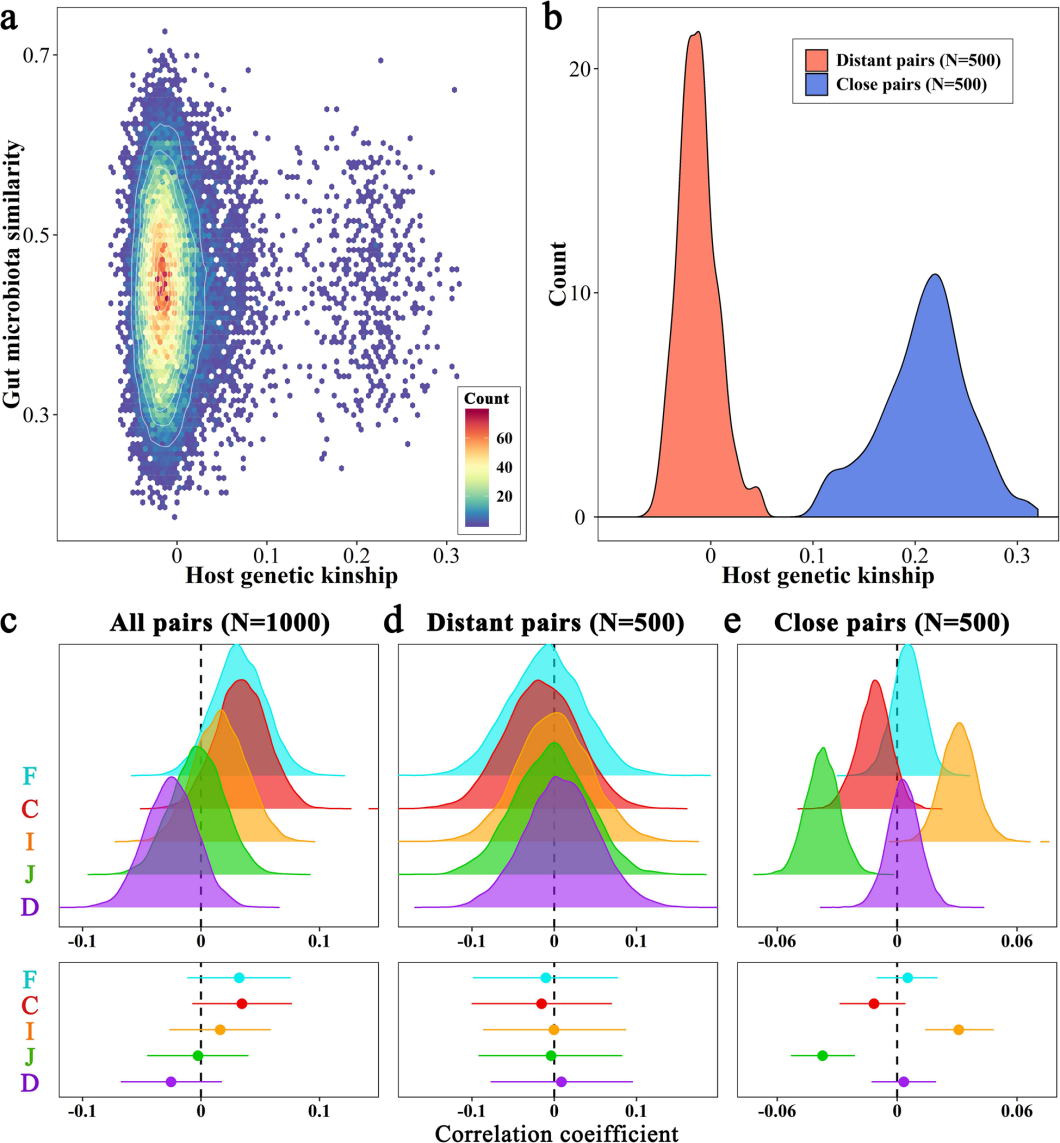
Relationships between host genetic kinship and gut microbial similarity. **a** Scatter plot of the host genetic kinship of pairs of individuals (*x* axis) and their microbial similarity based on the five sample types (*y* axis). **b** Distribution of host genetic kinship generated from random sampling. The pair of chickens with genetic kinship < 0.05 and > 0.10 are viewed as genetically more distant and close relatives, respectively. **c**–**e** The Pearson correlation coefficients (density plots) between host genetic kinship and the gut microbial similarity of 10,000 random sampling and their 95% confident intervals (bar plots). The dots in the bars denote the average of the correlation coefficients. D, J, I, C, and F represent the duodenum, jejunum, ileum, cecum and feces, respectively

Correlation analysis was performed on individuals for whom both genetic and microbial data were available. Given that most pairs of chickens showed no or a low degree of genetic relatedness (Fig. 4a), we randomly selected genetically more distant and close pairs of individuals (Fig. 4b) and calculated the correlations between host genetic kinship and Bray-Curtis similarity. This process was repeated 10,000 times, and the average correlation was − 0.025 for the duodenal microbiota, − 0.003 for the jejunal microbiota, 0.016 for the ileal microbiota, 0.034 for the cecal microbiota, and 0.032 for the fecal microbiota (Fig. 4c). The 95% confidence intervals of the correlation coefficients for the five sample types were − 0.068~0.018, − 0.046~0.040, − 0.027~0.059, − 0.007~0.077, and − 0.011~0.075, respectively. We separately calculated their association in genetically more distant and close pairs and obtained similar results: the correlations of genetic kinship with the gut microbial similarities within various sample types were close to zero, with a confidence interval of − 0.101~0.096 for the genetically more distant pairs group and − 0.053~0.048 for the genetically closer pairs group (Fig. 4d, e and Additional file 16: Figure S7a). In addition, no difference was found in the beta-diversities of the microbial communities between genetically more distant and close pairs (Additional file 16: Figure S7b).

**Fig. 5:**
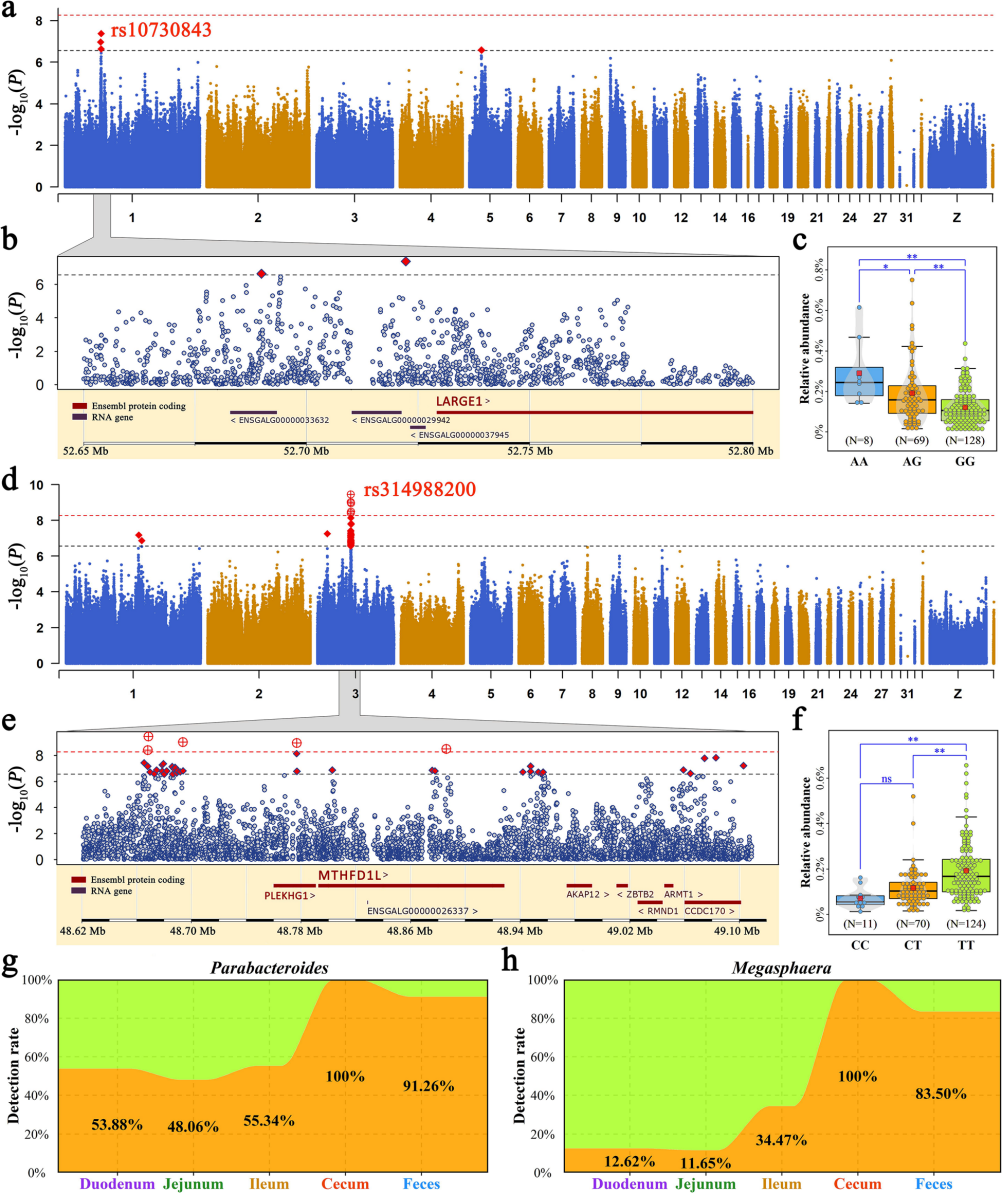
Microbial genome-wide association studies. **a**–**c** The relative abundance of the cecal *Parabacteroides* was associated with genetic variation near the gene *LARGE1*. **d**, **e** The relative abundance of the cecal *Megasphaera* was associated with genetic variation near and inside the gene *MTHFD1L*. **a**, **d** Genome-wide Manhattan plot: the horizontal red and black lines indicate genome-wide significance (*P* = 5.36 × 10^−9^) and suggestive significance (*P* = 2.78 × 10^−7^) thresholds. Each point represents an SNP. **b**, **c** Close-up plots of the 0.15- and 0.5-Mb windows around the SNP with the highest association, respectively. **c**, **f** Comparison of the relative abundance of the cecal microbiota among genotypes within the highest associated SNP locus. Each point represents a sample. The data and center red point indicate the number and mean value in the corresponding genotype, respectively. **, * and ns represent adjusted *P* values < 0.01, < 0.05, and > 0.05, respectively. Detection rates of **g** *Parabacteroides* and **h** *Megasphaera* in the five sampling sites

These results from correlation analysis implied that host genetics had a limited effect on the entire microbial community. However, this analysis was insufficient because host genetics may influence a small fraction of low-abundance microorganisms that contribute little to the entire community. Therefore, we performed GWAS on microbial genera and identified 6 and 44 genome-wide significant and suggestive significant loci associated with cecal *Parabacteroides* (Additional file 17: Table S9) and *Megasphaera* (Additional file 18: Table S10), respectively. The corresponding Q-Q plot for these GWAS is shown in Additional file 19: Figure S8. The most significant SNP controlling the relative abundance of *Parabacteroides* was rs10730843 (Fig. 5a). This SNP was located 5.1 kb upstream of the LARGE xylosyl- and glucuronyltransferase 1 (*LARGE1*, Fig. 5b) with an MAF of 0.22. The substitution of G to A for rs10730843 resulted in a significantly increased abundance of cecal *Parabacteroides* (Fig. 5c)*.* Regarding *Megasphaera*, 41 SNPs, located in a high LD region of 48.6~49.2 Mb on GGA3 were associated with the relative abundance of *Megasphaera* (Fig. 5d). As shown in Fig. 5e, seven genes presented this region. Among them, methylenetetrahydrofolate dehydrogenase (NADP+ dependent) 1 like (*MTHFD1L*) was the longest. The top significant SNP in this region was rs314988200. Chickens with the TT genotype of rs314988200 had a higher abundance of cecal *Megasphaera* (0.19%) than those with the CT and CC genotypes (0.12% and 0.07%, respectively; Fig. 5f). We further explored the spatial distribution of the two genera and found that *Parabacteroides* and *Megasphaera* mainly resided in the cecum, with a 100% detection rate (Fig. 5g, h), and their abundances in the cecum were not associated with those in the duodenum, jejunum, ileum, and feces.

### Proportion of variation in feed efficiency explained by host genetics and gut microbiota

**Fig. 6:**
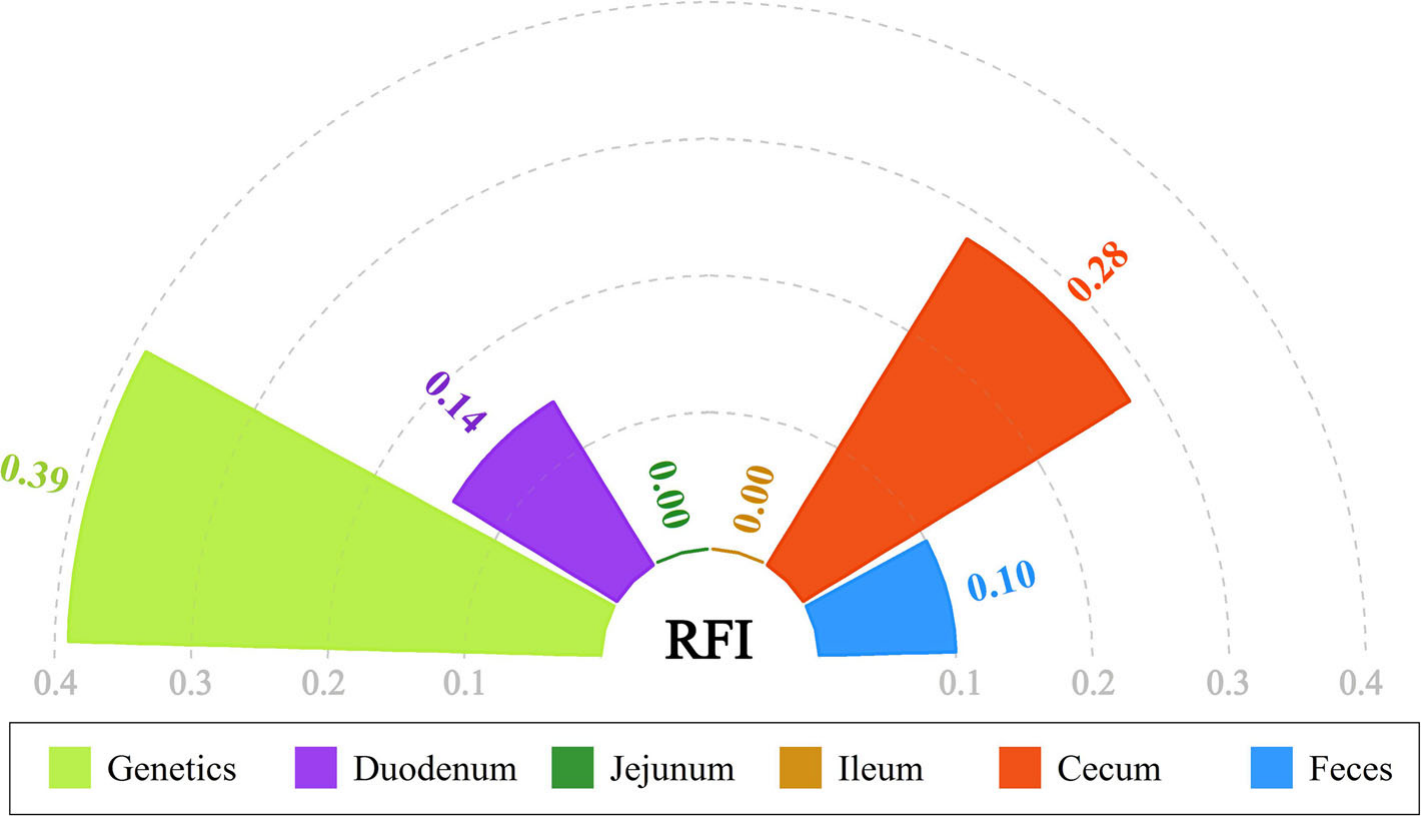
Heritability and microbiability of residual feed intake

The SNP-based *h*^2^ for RFI estimated in our study was 0.39, which was moderate, suggesting that host genetics played an important role in the regulation of feed efficiency. Analogous to heritability, the relative proportion of the total variance due to the gut microbial community is defined as the microbiability (*m*^2^), which allows for a holistic view of the effect of the microbiota on host traits. Larger *m*^2^ values indicate that the microbial community contributes more to the investigated phenotype. Because the microbial community within the digestive tract exhibits extensive spatial heterogeneity, we considered the microbial community that existed in diverse segments as different functional entities and dissected their relative contributions to feed efficiency. After correction for host genetics, the *m*^2^ of the RFI estimated based on the cecal microbiota was 0.28 (Fig. 6). However, the *m*^2^ values obtained from other anatomical sites were nonsignificant (0.14 for the duodenum, zero for the jejunum, and ileum, and 0.10 for the feces). These results indicated that the cecal microbiota was more closely associated with feed efficiency than the microbial communities from the duodenum, jejunum, ileum, and feces.

### Feed efficiency-related genetic variations and their effects on gut microbiota

**Fig. 7:**
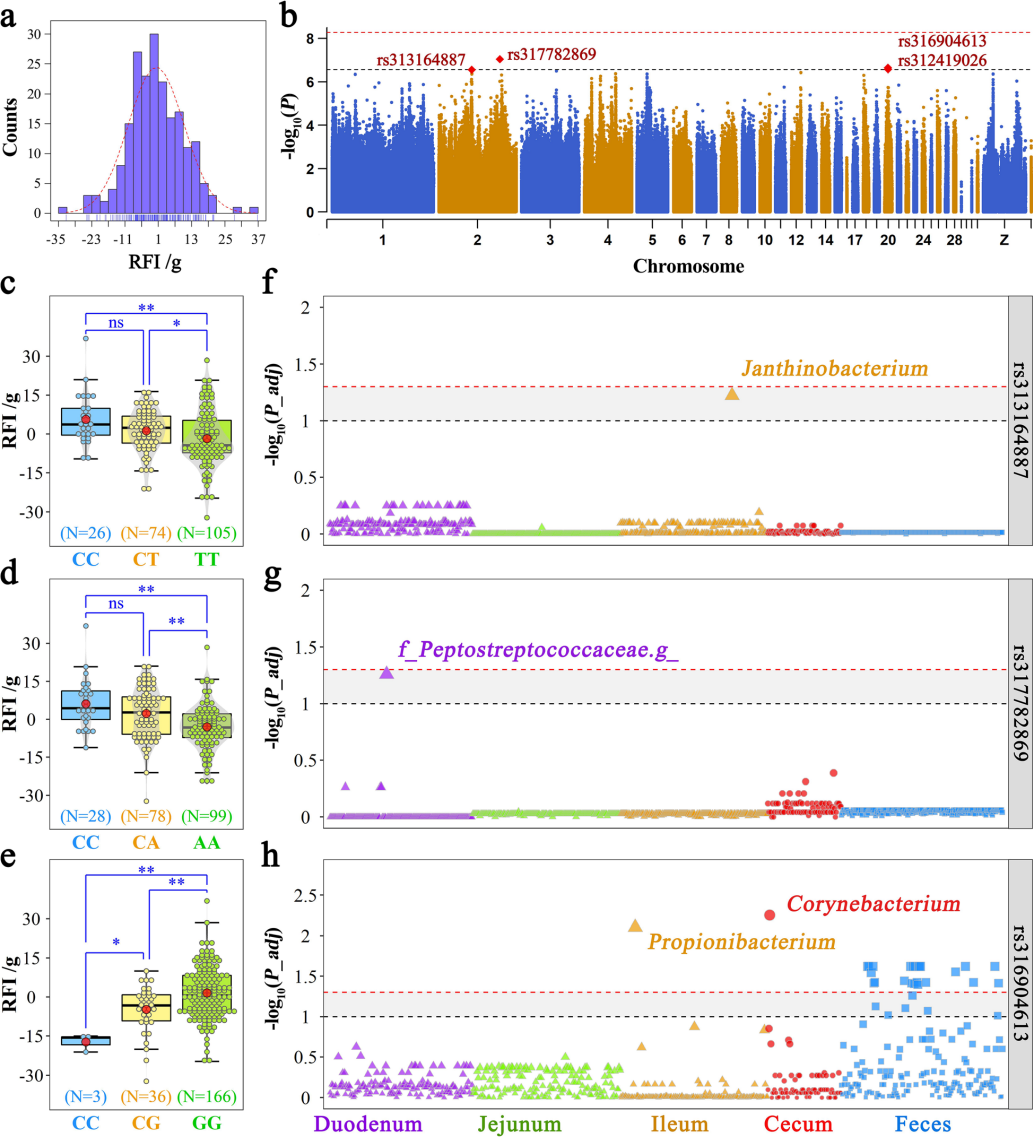
Effects of feed efficiency-related SNPs on the gut microbiota. **a** Distribution and **b** genome-wide association analysis for residual feed intake (RFI). The horizontal red and black lines indicate genome-wide significance (*P* = 5.36 × 10^−9^) and suggestive significance (*P* = 2.78 × 10^−7^) thresholds. **c**–**e** Comparison of RFI among genotypes within the RFI-related locus. Each point represents a chicken. The data and center red point indicate the number and mean value in the corresponding genotype, respectively. **, * and ns represent adjusted *P* values < 0.01, < 0.05, and > 0.05, respectively. **f**–**h** Comparison of RFI between genotypes within the RFI-related locus. Each point represents a chicken. The dashed red and gray lines indicate *P* value = 0.05 and 0.1, respectively

As described by the above results, host genetics had a prominent effect on the utilization of feed. Hence, the RFI was used as the feed efficiency phenotype for GWAS analysis, and RFI fitted a normal distribution (Fig. 7a). The corresponding Q-Q plot for the association is shown in Additional file 19: Figure S8. We found that 4 suggestive significant SNPs were related to RFI (Fig. 7b and Table 1). Among them, rs313164887 and rs312419026 resided in the intronic region of ELOVL fatty acid elongase 2 (*ELOVL2*) and phosphatidylinositol-3,4,5-trisphosphate-dependent Rac exchange factor 1 (*PREX1*), respectively. The other two variations, rs317782869 and rs316904613, were near the gene transient receptor potential cation channel subfamily A member 1 (*TRPA1*) and *PREX1*, respectively. In particular, rs316904613 and rs312419026 were highly linked to each other. At locus rs313164887, chickens with the TT genotype had a higher feed efficiency, with an RFI of − 1.80 g/day, than those with the CT and TT genotypes, with RFIs of 1.23 and 5.65 g/day, respectively (Fig. 7c). The variation in rs317782869 resulted from a base transversion (A/C). Birds with the major genotype (AA) were more feed efficient than those with the other two genotypes. The average RFIs for the AA, CA, and CC genotypes were − 3.00, 2.24, and 6.09 g/day, respectively (Fig. 7d). Regarding the SNP rs316904613, the G to C substitution led to a significant decrease in the RFI value (Fig. 7e).

**Table 1:** Detailed information on the SNPs associated with residual feed intake (RFI)

SNP	GGA	Position^a^	MAF^b^ (minor/major)	*P* value	Near gene	Gene name	Location
rs313164887	2	63367934 /63158673	0.31 (C/T)	2.78 × 10^−7^	ENSGALG00000012748	*ELOVL2*	Intronic
rs317782869	2	117906298 /117474376	0.33 (C/A)	9.08 × 10^−8^	ENSGALG00000031693	*KCNB2*	Intronic
					ENSGALG00000034751 (dist=157865)	*TRPA1*	–
rs316904613	20	6205401 /6013969	0.10 (C/G)	2.24 × 10^−7^	ENSGALG00000004593 (dist=38515)	*SULF2*	Intergenic
					ENSGALG00000004621 (dist=180107)	*PREX1*	
rs312419026	20	6450286 /6258855	0.10 (G/A)	2.64 × 10^−7^	ENSGALG00000004621	*PREX1*	Intronic

^a^The first and second coordinates are the position in the galGal5 and galGal6 genome assembly, respectively

^b^The allele frequency of the first listed marker

To further investigate the joint effects of the genotypes and microbiotas on feed efficiency, the differences in the abundance of each microbiota were analyzed among the different genotypes. Considering that these significant SNPs had a low frequency of minor genotypes in the current population, the Wilcoxon rank-sum test was performed on the taxon abundances between chickens with major and other genotypes. Ileal *Janthinobacterium* and duodenal unclassified *Peptostreptococcaceae* were close to the significance level at the SNPs of rs313164887 and rs317782869, receptively (Fig. 7f, g). Additionally, as many as 25 microbial genera, including ileal *Propionibacterium* and cecal *Corynebacterium*, reached the significance level at the rs316904613 locus (Fig. 7h).

### Feed efficiency-related microorganisms and their spatial distribution

**Fig. 8:**
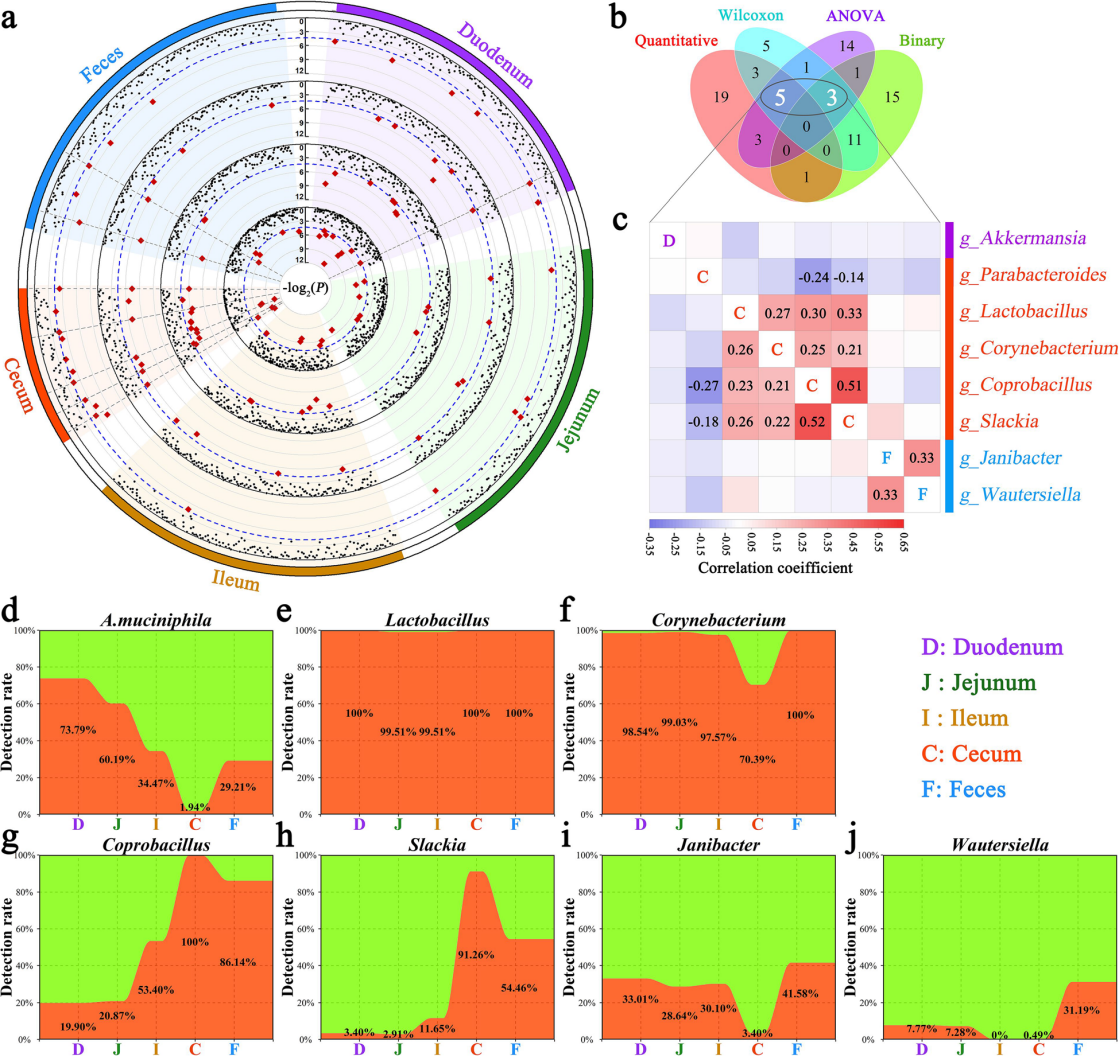
Identification of feed efficiency-associated microbiota. **a** Displayed from the outer to the inner circle are the sample type, Wilcoxon rank-sum test in each microbial abundance between the highest- and lowest-RFI chickens, ANOVA of RFI between the two groups with the highest and lowest microbial abundance, and association analysis using quantitative model and binary models, respectively. The adjusted *P* values for the significance test and association analysis are plotted as –log_2_(*P*). The dashed blue line represents *P* = 0.05. Each point represents a microbial genus, and the red point means that the genus passed the significance threshold. The dashed gray line indicates that the adjusted *P* values from the Wilcoxon rank-sum test, ANOVA, and any one of the association analyses are < 0.05. **b** Number of microbial genera associated with RFI detected by each test or association analysis and their overlaps. **c** Pearson’s (upper diagonal) and Spearman’s (lower diagonal) correlations among the shared 8 microbial genera. Significant *r* values are filled in numerically. **d**–**j** Detection rates of the RFI-related microbiota in the five sampling sites

Although host genetics could influence a small number of gut microorganisms, our results also revealed that host genetics and the gut microbiota contributed concurrently to feed efficiency in chickens. However, what are the microbial aspects governing this link? More specifically, can this impact be attributed to a specific taxon, or a combination of taxa? To answer this question, we performed a two-part model association analysis and two-tailed tests for the microbial genera and RFI. Thirty-one associations were detected by quantitative analysis, 31 associations were identified by binary analysis, and 28 and 27 genera were detected by the Wilcoxon-rank sum test and ANOVA, respectively (Fig. 8a). Among these associations, 8 genera were observed in both the association analysis and significance test (Fig. 8b). One (*Akkermansia*), five (*Parabacteroides*, *Lactobacillus*, *Corynebacterium*, *Coprobacillus*, and *Slackia*), and two (*Janibacter* and *Wautersiella*) of the shared genera were located in the duodenum, cecum, and feces, respectively. One was identified as *Akkermansia muciniphila* at the species level. Moreover, these cecal genera were positively and moderately correlated with each other (except for *Parabacteroides*; Fig. 8c).

Notably, *Parabacteroides* mainly resided in the cecum and was regulated by host genetics based on our aforementioned results. The detection rate of *A*. *muciniphila* incrementally decreased from 73.79 to 34.47% from the duodenum to the ileum (Fig. 8d), but *Akkermansia* was detected in less than 2% and 30% of the cecal and fecal samples, respectively. *Lactobacillus* was largely detected in any sampling site with a more than 99% detection rate (Fig. 8e). The genus *Corynebacterium* was detected in more than 97% of the duodenal, jejunal, ileal, and fecal samples, but its detection rate in the cecal sample was 70.39% (Fig. 8f). *Coprobacillus* and *Slackia* mainly resided in the cecum (Fig. 8g, h). The detection rates of both *Janibacter* and *Wautersiella* were both less than 35% in the small intestine and cecum (Fig. 8i, j).

**Fig. 9:**
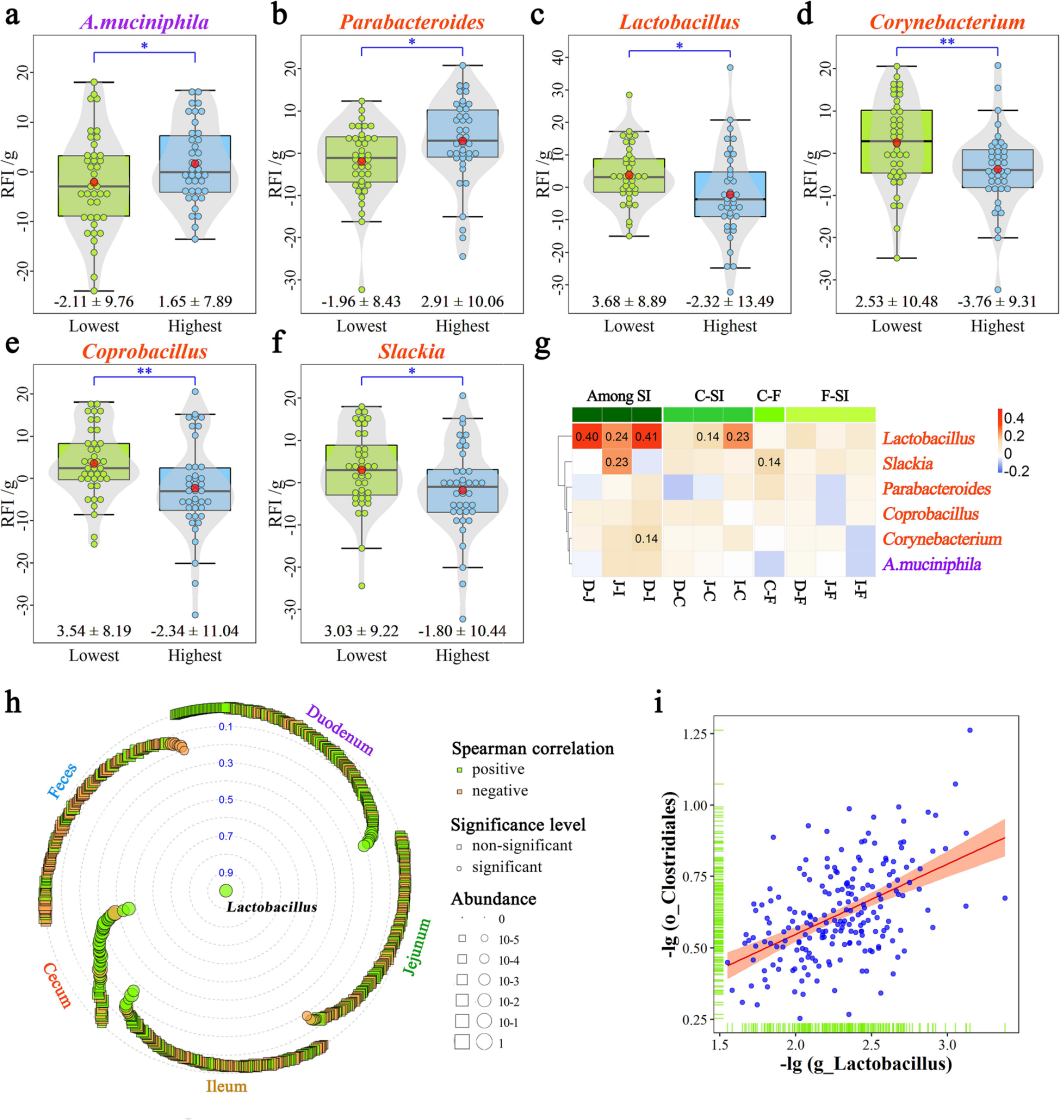
RFI-related microbiota and its association with other microbiota. Difference in RFI between the two groups with the highest and lowest abundance of **a** duodenal *A. muciniphila*, **b** cecal *Parabacteroides*, **c** cecal *Lactobacillus*, **d** cecal *Corynebacterium*, **e** cecal *Coprobacillus* and **f** cecal *Slackia*. Each point represents a sample. The center red point indicates the mean value in the corresponding group and the data are expressed as means ± SD. * and ** indicate adjusted *P*-values < 0.05 and 0.01. **g** Spearman’s correlation of specific microbiota among the five sampling sites. Red and blue tiles denote positive and negative correlations for each genus between two different sites, respectively, and a significant r-value is filled in numerically. SI, D, J, I, C and F indicate the small intestine, duodenum, jejunum, ileum, cecum and feces, respectively. **h** Spearman’s correlation between cecal *Lactobacillus* and other microbial genera (detected in at least 30% of samples in any of the five sample types). **i** Relationship between *Lactobacillus* and the order Clostridiales in the cecum

Given the low detection rate of two significant genera in fecal samples, we focused on the six most abundant microorganisms. The RFI value was significantly lower in the 20% of chickens with the lowest abundance of duodenal *Akkermansia* or cecal *Parabacteroides* than in the 20% with the highest abundance (Fig. 9a, b). Chickens with higher abundances of cecal *Lactobacillus*, *Corynebacterium*, *Coprobacillus*, and *Slackia* were more feed efficient than those with lower abundances of these microbial genera (Fig. 9c–f). Additionally, the genus *Lactobacillus* showed significant and positive correlations among the three segments of the small intestine, and between the ileum and cecum (Fig. 9g). Furthermore, cecal *Lactobacillus* exhibited significant and positive relationships with most of the cecal microorganisms (Fig. 9h), particularly the genera belonging to the order *Clostridiales* (Additional file 20: Table S11). The relationship between Clostridiales and *Lactobacillus* was 0.46 (Fig. 9i).

## Discussion

In recent years, a greater emphasis has been placed on improving feed efficiency in domestic animals. The efficiency of feed utilization is generally considered stable, but for poorly understood reasons, it varies considerably among individuals fed identical diets and reared under the same conditions. Host genetic variation is a key factor driving phenotypic variation. Additionally, emerging investigations have shown that variation in the gut microbiota may increase the phenotypic differences among individuals in a population [54–56]. The gut microbiome is considered the host second genome [14], is linked to feed digestion and nutrient absorption [15, 18, 57], inhibits the proliferation of intestinal pathogenic bacteria and stimulates the production of antimicrobial compounds [58, 59]. Despite these shared effects, whether host genetics shape and interact with the gut microbiota to influence feed efficiency in chickens is largely unknown. Clarifying the association between host genetics and gut microbiota in feed utilization is essential for the development of effective strategies to improve feed efficiency. Here, we used an automatic feeder to record the feed efficiency and performed whole-genome sequencing of hosts and 16S rRNA gene sequencing of the microbiota in 206 broiler chickens. The power of our present study is mainly reflected by the following factors. First, instead of focusing on single or a few parts of the gastrointestinal tract, we covered the chicken digestive tract segments more extensively, from the duodenum to the cloaca. Second, statistical models and methods of quantitative genetics were introduced in gut microbiota research to explore the relationship between host genetics and gut microbiota and to evaluate the influence of the microbial community on feed efficiency. Moreover, to our best knowledge, this is the largest association study in chickens linking the gut microbiota to feed efficiency to date.

The compartments within the digestive tract are differentiated from each other both morphologically and functionally. We and others have previously shown that their microbial composition is similarly distinct [33, 60, 61]. Microbial source tracking and phenotypic prediction further corroborated that the microbial communities differ markedly among the small intestine, cecum, and feces. Microorganisms engage in complex interactions with other organisms and their environment. Several microorganisms have a higher level of organization than individual cells termed biofilms, which are formed by multiple microbial populations embedded in complex, self-produced polymeric matrices that are adherent to each other and surfaces or interfaces [62]. Biofilm formation is an imperative strategy by which microbial communities survive and adapt to environments, particularly those with adverse conditions [63]. The higher biofilm formation ability of the small intestine communities, especially the duodenal microbiota, provides the mechanical properties necessary to protect the microbial communities from external forces such as digestive enzymes secreted by the host. Similar results based on microbial network inference also suggested a higher level of synergistic interactions in the small intestine. A clear negative correlation between anaerobic and aerobic bacteria was found in the ileum and may be caused by aerobe inhibition and anaerobe proliferation at lower oxygen concentrations. In the cecum, we observed a strong antagonistic relationship between Bacteroidales and Clostridiales, a finding that is consistent with the results of a study on human gut microbiome datasets [64]. Bacteroidetes and Clostridia represented most of the anaerobic fermentative bacteria [33], which may present nutritional competition for fermentation substrates in the cecum. These findings agree with the physiological structure of the chicken digestive tract. The heterogeneity of the gut sections urges caution in equating data from feces or a single gut compartment to data for the entire gut microbiota. Thus, we considered the microbial communities that existed in diverse sampling sites as different functional entities in the subsequent analysis.

Another fundamental goal of microbial research is to identify the factors that determine the gut microbial composition. Many environmental factors, such as diet [65] and geographic location [66], influence the gut microbiota of animals. However, the extent to which host genetic variation may play a role in determining microbial composition is debatable. Early studies in twins employed either culture or DNA fingerprinting-based techniques, and monozygotic siblings were found to have slightly more similar microbiomes than dizygotic siblings [67, 68]. The recent advent of sequence-based techniques has enabled gut microbiome studies with large cohorts. Several studies have revealed an increase in the overall similarity of gut microbial communities with greater degrees of relatedness between individuals [69–71], but this similarity decreased when siblings started living apart [72]. Compared with humans, domestic animals can be reared under controlled conditions. Previous studies have compared the differences in the gut microbiota between breeds. Pandit et al. [66] found cecal microbiota separation by chicken breeds or lines, whereas geographical location also exerted a substantial impact on the variation of cecal microbiota. In the present study, we found that the genetically closer pairwise individuals on average did not have more similar microbiota than genetically more distant pairs. We also demonstrated that the relationships between host genetic kinship and the gut microbial similarities within various sampling sites were weak. Similar results were also reported by Rothshild et al. [30] in human datasets and Massacci et al. [73] in horses. Certainly, other investigations in humans [74], mice [75], and pigs [71] have documented significant correlations between host genetic kinship and microbial similarity, while the correlation coefficient was weak, ranging from 0.14 to 0.19. These observations imply that most of the variation in the gut microbial community is due to factors other than host genetics.

Although studies examining the general measures of microbial similarity have not observed strong evidence for host genetic effects, a more general approach to this question has linked genetic loci with the gut microorganism abundance. A recent study identified several genetic variants involved in the immune response and metabolism that were significantly associated with microbial diversity in the cecum of chickens [32]. However, few studies in chicken have investigated the effect of the host on the abundance of a specific taxon. We published a study that identified a few heritable microbial taxa [33]. A natural next step is to pinpoint the host genetic variants and genes that underlie heritability. We performed GWAS in the present study and identified two genetic regions that were associated with the abundances of *Megasphaera* and *Parabacteroides* in the cecum, respectively. Previous studies have revealed that *Megasphaera* is a potent lactate utilizer in the rumen [16, 76] and plays an important role in preventing lactic acidosis [77, 78]. A candidate gene associated with cecal *Megasphaera* is *MTHFD1L*. Interestingly, Lee et al. [79] reported that lactate accumulated in *MTHFD1L*-knockdown cells, suggesting that *MTHFD1L* may interact with *Megasphaera* for lactate utilization. Regarding *Parabacteroides*, which have an intriguing association with glucolipid metabolism [80, 81], Wang et al. [81] demonstrated that oral treatment with live *Parabacteroides* reduced weight gain and improved glucose homeostasis. A promising candidate gene associated with the abundance of cecal *Parabacteroides* is *LARGE1*, which encodes a glycosyltransferase that may synthesize glycosphingolipid sugar chains. Because some gut microorganisms are partly under the control of host genes, from an animal breeder perspective, they can be considered host traits [82], highlighting the possibility of breeding for an optimized microbiota to indirectly improve feed efficiency. Indeed, *Parabacteroides* is one of the two more abundant genera in chickens with high RFI values (inefficient). Therefore, strategies to improve feed efficiency in chickens may be optimized by molecular breeding to decrease the abundance of cecal *Parabacteroides*.

In combination, microbial similarity and GWAS analyses implied that host genotypes interact with some microorganisms but cannot account for most microbial variation. Thus, the variation within the gut microbiota could be integrated into variance estimates of the host phenotype, a suggestion originally proposed by Ross et al. [83] and applied in humans [30], chickens [33], pigs [51], and cattle [52, 84]. Specifically, Camarinha-Silva et al. [51] found that the proportion of variance in feed efficiency traits explained by gut microbiota was higher than that explained by host genetics. To anticipate how much the efficiency of feed utilization could be modulated by host genetics and the gut microbiota in chickens, we estimated the *h*^2^ and *m*^2^ values of the RFI. Compared with the estimate generated using microbial information from one gut sample type, our estimates covered the chicken digestive tract contents more extensively, including the duodenum, jejunum, ileum, cecum, and feces. Host genetics was responsible for 39% of the total phenotypic variation. The effect of the microbial communities in the cecum on RFI was 28% after accounting for host genetics. These findings suggest agreement with the holobiont theory [85], where variations in the genome and microbiome can cause variations in host complex traits on which artificial selection and microbial regulation can act, such as selective breeding to unlock host genetic potential and feed supplementation with probiotics.

Consistent with early studies conducted with the same breed [11, 12], the RFI exhibited moderate heritability. This result indicated that feed efficiency was at a moderate level of genetic regulation. We then identified three suggestive significant loci—rs317782869, rs313164887, and rs316904613—which were near or distributed on three independent genes: *TRPA1*, *ELOVL2*, and *PREX1.* Among these, *TRPA1* encodes a protein known as transient receptor potential ankyrin 1, which is a member of the transient receptor potential channel family. Previous studies have demonstrated TRPA1 channel involvement in the regulation of gastrointestinal motility [86] and feed digestion [87] by 5-hydroxytryptamine and cholecystokinin release, respectively. In addition, this channel plays a crucial role in the pathogenesis of inflammatory bowel disease [88]. The *ELOVL2* gene encodes a transmembrane protein that is involved in long-chain polyunsaturated fatty acid elongation and lipid synthesis [89–91]. Lipids are the principal stored forms of energy in many organisms. Mouse *ELOVL2* is mainly expressed in the liver and testicle [92], while Gregory et al. [90] found that the expression level of *ELOVL2* was 4.6-fold in the liver in chickens compared with that in the mouse [93]. Jehl et al. [94] found that a low-energy diet led to overexpression of the *ELOVL2* gene. The *PREX1* gene was also found to be significantly associated with feed efficiency in cattle [95]. Previous studies have reported that PREX1 is involved in the thermogenic capacity and insulin-stimulated glucose uptake in adipocytes [96, 97]. These observations suggested that the *TRPA1* gene may influence feed efficiency by regulating nutrient digestion, while *ELOVL2* and *PREX1* may affect energy utilization and, consequently, feed efficiency. A significant difference in RFI was found among chicken individuals differing at specific genetic loci: rs317782869, rs313164887, and rs316904613. Moreover, a subset of microbial genera, particularly *Janthinobacterium*, *Propionibacterium*, and *Corynebacterium,* reached the significance level between chickens with various genotypes. Munyaka et al. [98] observed an increase in *Janthinobacterium* in the ileum with a corn-based diet. *Propionibacterium* is a Gram-positive bacterium with a unique ability to produce propionate [99] and is related to higher energy efficiency [100]. Members of the genus *Corynebacterium* metabolize various carbohydrates and produce organic acids such as lactate and succinate [101]. Moreover, *Corynebacterium* is one of the six genera identified as being associated with feed utilization. These findings indicated that breeding for high feed efficiency by targeting source variation could also influence a small percentage of the gut microbiota, thereby together contributing to the variation in feed efficiency.

The microbiability of RFI estimated for the cecal microbiota was 0.28, while the proportion of variance in RFI explained by the duodenal, jejunal, ileal, and fecal microbiota was statistically insignificant. Moreover, of the six genera that were significantly associated with RFI in our study, five were located in the cecum. Thus, the contribution of the cecal microbiota to feed efficiency was higher than that of other parts of the intestinal tract. Stanley et al. [102] compared the jejunal and cecal microbiota between chickens with high and low feed efficiency and found that 24 cecal microorganisms were significantly differentially abundant between the two groups, but the jejunal microbial communities showed no difference. Our previous study in egg-type chickens also showed that the microbial communities in the cecum were significantly different between the high- and low-RFI groups, while no clear separation was found in the duodenal or fecal microbial communities between the two groups [54]. The cecum is a highly anaerobic environment. Numerous polysaccharide- and oligosaccharide-degrading enzyme-encoding genes are found in the cecal metagenome [103]. The cecum can ferment indigestible ingredients into energy-rich SCFAs. Annison et al. [104] demonstrated that the cecum is the main production site of SCFAs in chickens. Moreover, the almost complete absence of SCFAs in the digestive tract contents of germ-free chickens [104] implies that the SCFAs normally present in the tract are of microbial origin. SCFAs produced by microbial fermentation within the cecum increase the absorption and utilization of energy by the host. Previous studies have revealed that cecal SCFAs could provide up to 11~18% of the energy needs for the basal metabolic rate and 4~7% of the estimated free-living energy requirements [105, 106].

Given that the resident microbiota affects the efficiency of feed utilization, we further explored which taxa were significantly linked to feed efficiency. Our study confirmed that lower abundances of duodenal *A*. *muciniphila* and cecal *Parabacteroides* and higher abundances of cecal *Lactobacillus*, *Corynebacterium*, *Coprobacillus*, and *Slackia* were associated with better feed efficiency. *A*. *muciniphila*, *Parabacteroides*, and *Lactobacillus* are known to be involved in feed efficiency. The other three bacteria we identified are novel findings. Several studies have reported positive correlations between the abundance of *A*. *muciniphila* and energy expenditure or thermogenesis [107–109]. Interestingly, a recent study confirmed that daily oral administration of pasteurized *A*. *muciniphila* increase energy excretion in the feces and decrease food energy efficiency [110]. *Parabacteroides*, as mentioned above, is important in the regulation of host metabolic functions [80, 81] and is regulated by host genetics. *Lactobacillus* is currently recommended as a probiotic to improve production performance in poultry production; our previous study also showed that the abundance of cecal *Lactobacillus* was significantly higher in hens with better feed efficiency [54]. *Lactobacillus* can inhabit various sections of the chicken digestive tract. The relative abundances of *Lactobacillus* in adjacent sites were similar; specifically, a positive correlation was detected. Altaher et al. [111] found that chicken feed efficiency was improved by 6.4% through dietary supplementation with *Lactobacillus*. Similar results were also reported by Gao et al. [112]. Additionally, we found that cecal *Lactobacillus* exhibited significant and positive relationships with most of cecal microorganisms, especially those of genera belonging to the order Clostridiales, which are the main SCFA-producing bacteria in chickens. Supplementation with *Lactobacillus* increased the abundances of many intestinal *Lactobacillus* spp. and promoted a beneficial change in the bacterial correlation network [112].

## Conclusions

Our study strengthens the notion that both host genetic and gut microbial variations can lead to variation in feed efficiency. Overall, gut microbial similarity was largely independent of individual genetic relatedness. However, a small number of microorganisms could interact with host genotypes and were also linked to feed efficiency. We further identified three independent SNPs that were associated with feed efficiency and had a modest effect on the gut microbiota. These results revealed host-microbiota interactions in the regulation of feed efficiency. Gut microbial communities among the compartments of the digestive tract exhibited substantial spatial heterogeneity, and the contributions of the gut microbiota to RFI varied along the digestive tract. Among these, the cecal microbiota had a much larger effect on feed efficiency. Additionally, six bacteria, *Akkermansia muciniphila*, *Parabacteroides*, *Lactobacillus*, *Corynebacterium*, *Coprobacillus*, and *Slackia*, were identified for their significant associations with feed efficiency. These observations collectively provide insights into the linkage between the gut microbiota and host genetics regarding chicken feed efficiency and may aid in developing strategies to improve feed efficiency in chickens.

## Supplementary Information

**Additional file 1: Table S1.** Composition of the diets for chickens during the experiment.

**Additional file 2: Table S2.** Descriptive statistics for host phenotypes.

**Additional file 3: Figure S1.** Distribution and correlation of host phenotypes.

**Additional file 4: Figure S2.** Collection of blood from the wing vein and the sampling sites of the gut content and feces.

**Additional file 5: Figure S3.** The principal component analysis of the host genetics.

**Additional file 6: Table S3.** Statistics of SNPs in functional regions.

**Additional file 7: Text S1.** Sequence processes and quality control pipeline.

**Additional file 8: Figure S4.** Decay of chromosome-wide linkage disequilibrium (LD).

**Additional file 9: Figure S5.** Workflow of the two-part model (cited from Fu et al. [53]).

**Additional file 10: Figure S6.** Differences in the relative abundance of facultative anaerobes within the four gut segments and feces.

**Additional file 11: Table S4.** Co-occurrence network of duodenal microbial taxa.

**Additional file 12: Table S5.** Co-occurrence network of jejunal microbial taxa.

**Additional file 13: Table S6.** Co-occurrence network of ileal microbial taxa.

**Additional file 14: Table S7.** Co-occurrence network of cecal microbial taxa.

**Additional file 15: Table S8.** Co-occurrence network of fecal microbial taxa.

**Additional file 16: Figure S7.** Comparison of the gut microbial similarity between genetically distinct relatives. (a) Correlations between host genetic kinship and the Bray-Curtis distance in the five sampling sites. (b) Comparison of the Bray-Curtis distance between more distant and close relatives.

**Additional file 17: Table S9.** Detailed information on the SNPs associated with the abundance of cecal *Parabacteroides.*

**Additional file 18: Table S10.** Detailed information on the SNPs associated with the abundance of cecal *Megasphaera.*

**Additional file 19: Figure S8.** The corresponding Q-Q plots for the genome-wide associations studies.

**Additional file 20: Table S11.** Spearman’s correlation between cecal *Lactobacillus* and other microbial genera.

## Data Availability

The raw data are available from the Sequence Read Archive with accession numbers PRJNA449436, PRJNA449437, and PRJNA449438.

## Abbreviations

ADFI: Average daily feed intake
ADG: Average daily gain
ANOVA: Analysis of variance
BH: Benjamini–Hochberg
*ELOVL2*: ELOVL fatty acid elongase 2
GWAS: Genome-wide association study
h^2^: Heritability
*LARGE1*: LARGE xylosyl- and glucuronyltransferase 1
LD: Linkage disequilibrium
MAF: Minor allele frequency
MRPP: Multi-response permutation procedure
*MTHFD1L*: Methylenetetrahydrofolate dehydrogenase (NADP+ dependent) 1 like
m^2^: Microbiability
NMDS: Nonmetric multidimensional scaling
OTUs: Operational taxonomic units
*PREX1*: Phosphatidylinositol-3,4,5-trisphosphate dependent Rac exchange factor 1
RFI: Residual feed intake
SCFAs: Short-chain fatty acids
SNPs: Single-nucleotide polymorphisms
*SULF2*: Sulfatase 2
*TRPA1*: Transient receptor potential cation channel subfamily A member 1

## References

[1] Keeton JT, Dikeman ME (2017). 'Red' and 'white' meats—terms that lead to confusion. Anim Front..

[2] Mottet A, Tempio G (2017). Global poultry production: current state and future outlook and challenges. Worlds Poult Sci J..

[3] Brameld JM, Parr T (2016). Improving efficiency in meat production. Proc Nutr Soc..

[4] Godfray HC, Beddington JR, Crute IR, Haddad L, Lawrence D, Muir JF (2010). Food security: the challenge of feeding 9 billion people. Science..

[5] Hill J, Nelson E, Tilman D, Polasky S, Tiffany D (2006). Environmental, economic, and energetic costs and benefits of biodiesel and ethanol biofuels. Proc Natl Acad Sci U S A..

[6] Tigchelaar M, Battisti DS, Naylor RL, Ray DK (2018). Future warming increases probability of globally synchronized maize production shocks. Proc Natl Acad Sci U S A..

[7] Wen C, Yan W, Zheng J, Ji C, Zhang D, Sun C, Yang N (2018). Feed efficiency measures and their relationships with production and meat quality traits in slower growing broilers. Poult Sci..

[8] Zhang W, Aggrey SE (2003). Genetic variation in feed utilization efficiency of meat-type chickens. Worlds Poult Sci J..

[9] Aggrey SE, Karnuah AB, Sebastian B, Anthony NB (2010). Genetic properties of feed efficiency parameters in meat-type chickens. Genet Sel Evol..

[10] Rekaya R, Sapp RL, Wing T, Aggrey SE (2013). Genetic evaluation for growth, body composition, feed efficiency, and leg soundness. Poult Sci..

[11] Xu Z, Ji C, Zhang Y, Zhang Z, Nie Q, Xu J, Zhang D, Zhang X (2016). Combination analysis of genome-wide association and transcriptome sequencing of residual feed intake in quality chickens. BMC Genomics..

[12] Ye S, Chen Z, Zheng R, Diao S, Teng J, Yuan X (2020). New insights from imputed whole-genome sequence-based genome-wide association analysis and transcriptome analysis: the genetic mechanisms underlying residual feed intake in chickens. Front Genet..

[13] Zuidhof MJ, Schneider BL, Carney VL, Korver DR, Robinson FE (2014). Growth, efficiency, and yield of commercial broilers from 1957, 1978, and 2005. Poult Sci..

[14] Grice EA, Segre JA (2012). The human microbiome: our second genome. Annu Rev Genom Hum G..

[15] Turnbaugh PJ, Ley RE, Mahowald MA, Magrini V, Mardis ER, Gordon JI (2006). An obesity-associated gut microbiome with increased capacity for energy harvest. Nature..

[16] Shabat SK, Sasson G, Doron-Faigenboim A, Durman T, Yaacoby S, Berg MM (2016). Specific microbiome-dependent mechanisms underlie the energy harvest efficiency of ruminants. Isme J..

[17] Martinez-Guryn K, Hubert N, Frazier K, Urlass S, Musch MW, Ojeda P, Pierre JF, Miyoshi J, Sontag TJ, Cham CM, Reardon CA, Leone V, Chang EB (2018). Small intestine microbiota regulate host digestive and absorptive adaptive responses to dietary lipids. Cell Host Microbe..

[18] Tremaroli V, Bäckhed F (2012). Functional interactions between the gut microbiota and host metabolism. Nature..

[19] Koh A, De Vadder F, Kovatcheva-Datchary P, Bäckhed F (2016). From dietary fiber to host physiology: Short-chain fatty acids as key bacterial metabolites. Cell..

[20] Stanley D, Hughes RJ, Geier MS, Moore RJ (2016). Bacteria within the gastrointestinal tract microbiota correlated with improved growth and feed conversion: challenges presented for the identification of performance enhancing probiotic bacteria. Front Microbiol.

[21] Siegerstetter S, Schmitz-Esser S, Magowan E, Wetzels SU, Zebeli Q, Lawlor PG (2017). Intestinal microbiota profiles associated with low and high residual feed intake in chickens across two geographical locations. PLoS One..

[22] Metzler-Zebeli BU, Siegerstetter SC, Magowan E, Lawlor PG, Petri RM, O CN (2019). Feed restriction modifies intestinal microbiota-host mucosal networking in chickens divergent in residual feed intake. mSystems.

[23] Mignon-Grasteau S, Narcy A, Rideau N, Chantry-Darmon C, Boscher M, Sellier N (2015). Impact of selection for digestive efficiency on microbiota composition in the chicken. PLoS One..

[24] Borey M, Estellé J, Caidi A, Bruneau N, Coville J, Hennequet-Antier C (2020). Broilers divergently selected for digestibility differ for their digestive microbial ecosystems. PLoS One..

[25] Li F, Li C, Chen Y, Liu J, Zhang C, Irving B, Fitzsimmons C, Plastow G, Guan LL (2019). Host genetics influence the rumen microbiota and heritable rumen microbial features associate with feed efficiency in cattle. Microbiome..

[26] Sasson G, Kruger BS, Seroussi E, Doron-Faigenboim A, Shterzer N, Yaacoby S (2017). Heritable bovine rumen bacteria are phylogenetically related and correlated with the cow's capacity to harvest energy from its feed. mBio.

[27] Org E, Parks BW, Joo JWJ, Emert B, Schwartzman W, Kang EY, Mehrabian M, Pan C, Knight R, Gunsalus R, Drake TA, Eskin E, Lusis AJ (2015). Genetic and environmental control of host-gut microbiota interactions. Genome Res..

[28] Goodrich JK, Davenport ER, Beaumont M, Jackson MA, Knight R, Ober C, Spector TD, Bell JT, Clark AG, Ley RE (2016). Genetic determinants of the gut microbiome in UK twins. Cell Host Microbe..

[29] Wang J, Thingholm LB, Skiecevičienė J, Rausch P, Kummen M, Hov JR, Degenhardt F, Heinsen FA, Rühlemann MC, Szymczak S, Holm K, Esko T, Sun J, Pricop-Jeckstadt M, al-Dury S, Bohov P, Bethune J, Sommer F, Ellinghaus D, Berge RK, Hübenthal M, Koch M, Schwarz K, Rimbach G, Hübbe P, Pan WH, Sheibani-Tezerji R, Häsler R, Rosenstiel P, D'Amato M, Cloppenborg-Schmidt K, Künzel S, Laudes M, Marschall HU, Lieb W, Nöthlings U, Karlsen TH, Baines JF, Franke A (2016). Genome-wide association analysis identifies variation in vitamin D receptor and other host factors influencing the gut microbiota. Nat Genet..

[30] Rothschild D, Weissbrod O, Barkan E, Kurilshikov A, Korem T, Zeevi D, Costea PI, Godneva A, Kalka IN, Bar N, Shilo S, Lador D, Vila AV, Zmora N, Pevsner-Fischer M, Israeli D, Kosower N, Malka G, Wolf BC, Avnit-Sagi T, Lotan-Pompan M, Weinberger A, Halpern Z, Carmi S, Fu J, Wijmenga C, Zhernakova A, Elinav E, Segal E (2018). Environment dominates over host genetics in shaping human gut microbiota. Nature..

[31] Bergamaschi M, Maltecca C, Schillebeeckx C, McNulty NP, Schwab C, Shull C (2020). Heritability and genome-wide association of swine gut microbiome features with growth and fatness parameters. Sci Rep..

[32] Psifidi A, Crotta M, Pandit R, Fosso B, Koringa P, Limon G, et al. Identification of SNP markers associated with gut microbiome composition in chicken. In: Proceedings of the world congress on genetics applied to livestock production. New Zealand: WCGALP Archive; 2018. p. 584.

[33] Wen C, Yan W, Sun C, Ji C, Zhou Q, Zhang D, Zheng J, Yang N (2019). The gut microbiota is largely independent of host genetics in regulating fat deposition in chickens. ISME J..

[34] Martinez-Guryn K, Leone V, Chang EB (2019). Regional diversity of the gastrointestinal microbiome. Cell Host Microbe..

[35] Yan W, Sun C, Wen C, Ji C, Zhang D, Yang N (2019). Relationships between feeding behaviors and performance traits in slow-growing yellow broilers. Poult Sci..

[36] Li H, Durbin R (2009). Fast and accurate short read alignment with Burrows-Wheeler transform. Bioinformatics..

[37] McKenna A, Hanna M, Banks E, Sivachenko A, Cibulskis K, Kernytsky A, Garimella K, Altshuler D, Gabriel S, Daly M, DePristo MA (2010). The genome analysis toolkit: a MapReduce framework for analyzing next-generation DNA sequencing data. Genome Res..

[38] Wang K, Li M, Hakonarson H (2010). ANNOVAR: Functional annotation of genetic variants from high-throughput sequencing data. Nucleic Acids Res..

[39] Caporaso JG, Kuczynski J, Stombaugh J, Bittinger K, Bushman FD, Costello EK, Fierer N, Peña AG, Goodrich JK, Gordon JI, Huttley GA, Kelley ST, Knights D, Koenig JE, Ley RE, Lozupone CA, McDonald D, Muegge BD, Pirrung M, Reeder J, Sevinsky JR, Turnbaugh PJ, Walters WA, Widmann J, Yatsunenko T, Zaneveld J, Knight R (2010). QIIME allows analysis of high-throughput community sequencing data. Nat Methods..

[40] Magoc T, Salzberg SL (2011). FLASH: fast length adjustment of short reads to improve genome assemblies. Bioinformatics..

[41] Quast C, Pruesse E, Yilmaz P, Gerken J, Schweer T, Yarza P, Peplies J, Glöckner FO (2012). The SILVA ribosomal RNA gene database project: improved data processing and web-based tools. Nucleic Acids Res..

[42] Shenhav L, Thompson M, Joseph TA, Briscoe L, Furman O, Bogumil D, Mizrahi I, Pe’er I, Halperin E (2019). FEAST: fast expectation-maximization for microbial source tracking. Nat Methods..

[43] Ward T, Larson J, Meulemans J, Hillmann B, Lynch J, Sidiropoulos D, et al. BugBase predicts organism level microbiome phenotypes. bioRxiv. 2017:133462. 10.1101/133462.

[44] Shannon P, Markiel A, Ozier O, Baliga NS, Wang JT, Ramage D, Amin N, Schwikowski B, Ideker T (2003). Cytoscape: a software environment for integrated models of biomolecular interaction networks. Genome Res..

[45] Igartua C, Davenport ER, Gilad Y, Nicolae DL, Pinto J, Ober C (2017). Host genetic variation in mucosal immunity pathways influences the upper airway microbiome. Microbiome..

[46] Hughes DA, Bacigalupe R, Wang J, Rühlemann MC, Tito RY, Falony G, Joossens M, Vieira-Silva S, Henckaerts L, Rymenans L, Verspecht C, Ring S, Franke A, Wade KH, Timpson NJ, Raes J (2020). Genome-wide associations of human gut microbiome variation and implications for causal inference analyses. Nat Microbiol..

[47] Zhou X, Stephens M (2012). Genome-wide efficient mixed-model analysis for association studies. Nat Genet..

[48] Gao X, Starmer J, Martin ER (2008). A multiple testing correction method for genetic association studies using correlated single nucleotide polymorphisms. Genet Epidemiol..

[49] Purcell S, Neale B, Todd-Brown K, Thomas L, Ferreira MA, Bender D (2007). PLINK: a tool set for whole-genome association and population-based linkage analyses. Am J Hum Genet..

[50] Yang J, Lee SH, Goddard ME, Visscher PM (2011). GCTA: A tool for genome-wide complex trait analysis. Am J Hum Genet..

[51] Camarinha-Silva A, Maushammer M, Wellmann R, Vital M, Preuss S, Bennewitz J (2017). Host genome influence on gut microbial composition and microbial prediction of complex traits in pigs. Genetics..

[52] Difford GF, Plichta DR, Løvendahl P, Lassen J, Noel SJ, Højberg O, Wright ADG, Zhu Z, Kristensen L, Nielsen HB, Guldbrandtsen B, Sahana G (2018). Host genetics and the rumen microbiome jointly associate with methane emissions in dairy cows. PLoS Genet..

[53] Fu J, Bonder MJ, Cenit MC, Tigchelaar-Feenstra E, Maatman A, Dekens JAM (2015). The gut microbiome contributes to a substantial proportion of the variation in blood lipids. Circ Res..

[54] Yan W, Sun C, Yuan J, Yang N (2017). Gut metagenomic analysis reveals prominent roles of *Lactobacillus* and cecal microbiota in chicken feed efficiency. Sci Rep..

[55] Shah TM, Patel JG, Gohil TP, Blake DP, Joshi CG (2019). Host transcriptome and microbiome interaction modulates physiology of full-sibs broilers with divergent feed conversion ratio. NPJ Biofilms Microbi..

[56] Li F, Hitch T, Chen Y, Creevey CJ, Guan LL (2019). Comparative metagenomic and metatranscriptomic analyses reveal the breed effect on the rumen microbiome and its associations with feed efficiency in beef cattle. Microbiome..

[57] Rubino F, Carberry C, Waters SM, Kenny D, McCabe MS, Creevey CJ (2017). Divergent functional isoforms drive niche specialisation for nutrient acquisition and use in rumen microbiome. ISME J..

[58] Fanning S, Hall LJ, Cronin M, Zomer A, MacSharry J, Goulding D, O'Connell Motherway M, Shanahan F, Nally K, Dougan G, van Sinderen D (2012). Bifidobacterial surface-exopolysaccharide facilitates commensal-host interaction through immune modulation and pathogen protection. Proc Natl Acad Sci U S A..

[59] Hooper LV (2009). Do symbiotic bacteria subvert host immunity?. Nat Rev Microbiol..

[60] Huang P, Zhang Y, Xiao K, Jiang F, Wang H, Tang D, Liu D, Liu B, Liu Y, He X, Liu H, Liu X, Qing Z, Liu C, Huang J, Ren Y, Yun L, Yin L, Lin Q, Zeng C, Su X, Yuan J, Lin L, Hu N, Cao H, Huang S, Guo Y, Fan W, Zeng J (2018). The chicken gut metagenome and the modulatory effects of plant-derived benzylisoquinoline alkaloids. Microbiome..

[61] Grond K, Guilani H, Hird SM (2020). Spatial heterogeneity of the shorebird gastrointestinal microbiome. Roy Soc Open Sci..

[62] Flemming HC, Wuertz S (2019). Bacteria and archaea on Earth and their abundance in biofilms. Nat Rev Microbiol..

[63] Yan J, Bassler BL (2019). Surviving as a community: antibiotic tolerance and persistence in bacterial biofilms. Cell Host Microbe..

[64] Ramanan D, Bowcutt R, Lee SC, Tang MS, Kurtz ZD, Ding Y, Honda K, Gause WC, Blaser MJ, Bonneau RA, Lim YAL, Loke P’, Cadwell K (2016). Helminth infection promotes colonization resistance via type 2 immunity. Science..

[65] David LA, Maurice CF, Carmody RN, Gootenberg DB, Button JE, Wolfe BE, Ling AV, Devlin AS, Varma Y, Fischbach MA, Biddinger SB, Dutton RJ, Turnbaugh PJ (2014). Diet rapidly and reproducibly alters the human gut microbiome. Nature..

[66] Pandit RJ, Hinsu AT, Patel NV, Koringa PG, Jakhesara SJ, Thakkar JR, Shah TM, Limon G, Psifidi A, Guitian J, Hume DA, Tomley FM, Rank DN, Raman M, Tirumurugaan KG, Blake DP, Joshi CG (2018). Microbial diversity and community composition of caecal microbiota in commercial and indigenous Indian chickens determined using 16s rDNA amplicon sequencing. Microbiome..

[67] Stewart JA (2005). Investigations into the influence of host genetics on the predominant eubacteria in the faecal microflora of children. J Med Microbiol..

[68] Van de Merwe JP, Stegeman JH, Hazenberg MP (1983). The resident faecal flora is determined by genetic characteristics of the host. Implications for Crohn's disease?. Antonie Van Leeuwenhoek..

[69] Yatsunenko T, Rey FE, Manary MJ, Trehan I, Dominguez-Bello MG, Contreras M, Magris M, Hidalgo G, Baldassano RN, Anokhin AP, Heath AC, Warner B, Reeder J, Kuczynski J, Caporaso JG, Lozupone CA, Lauber C, Clemente JC, Knights D, Knight R, Gordon JI (2012). Human gut microbiome viewed across age and geography. Nature..

[70] Goodrich JK, Waters JL, Poole AC, Sutter JL, Koren O, Blekhman R, Beaumont M, van Treuren W, Knight R, Bell JT, Spector TD, Clark AG, Ley RE (2014). Human genetics shape the gut microbiome. Cell..

[71] Chen C, Huang X, Fang S, Yang H, He M, Zhao Y, Huang L (2018). Contribution of host genetics to the variation of microbial composition of cecum lumen and feces in pigs. Front Microbiol..

[72] Xie H, Guo R, Zhong H, Feng Q, Lan Z, Qin B, Ward KJ, Jackson MA, Xia Y, Chen X, Chen B, Xia H, Xu C, Li F, Xu X, al-Aama JY, Yang H, Wang J, Kristiansen K, Wang J, Steves CJ, Bell JT, Li J, Spector TD, Jia H (2016). Shotgun metagenomics of 250 adult twins reveals genetic and environmental impacts on the gut microbiome. Cell Syst..

[73] Massacci FR, Clark A, Ruet A, Lansade L, Costa M, Mach N (2020). Inter-breed diversity and temporal dynamics of the faecal microbiota in healthy horses. J Anim Breed Genet..

[74] Blekhman R, Goodrich JK, Huang K, Sun Q, Bukowski R, Bell JT, Spector TD, Keinan A, Ley RE, Gevers D, Clark AG (2015). Host genetic variation impacts microbiome composition across human body sites. Genome Biol..

[75] Suzuki TA, Phifer-Rixey M, Mack KL, Sheehan MJ, Lin D, Bi K, Nachman MW (2019). Host genetic determinants of the gut microbiota of wild mice. Mol Ecol..

[76] Kamke J, Kittelmann S, Soni P, Li Y, Tavendale M, Ganesh S, Janssen PH, Shi W, Froula J, Rubin EM, Attwood GT (2016). Rumen metagenome and metatranscriptome analyses of low methane yield sheep reveals a Sharpea-enriched microbiome characterised by lactic acid formation and utilisation. Microbiome..

[77] Plaizier JC, Li S, Tun HM, Khafipour E (2017). Nutritional models of experimentally-induced subacute ruminal acidosis (SARA) differ in their impact on rumen and hindgut bacterial communities in dairy cows. Front Microbiol..

[78] Klieve AV, Hennessy D, Ouwerkerk D, Forster RJ, Mackie RI, Attwood GT (2003). Establishing populations of *Megasphaera elsdenii* YE 34 and *Butyrivibrio fibrisolvens* YE 44 in the rumen of cattle fed high grain diets. J Appl Microbiol..

[79] Lee D, Xu IM, Chiu DK, Lai RK, Tse AP, Lan LL (2017). Folate cycle enzyme MTHFD1L confers metabolic advantages in hepatocellular carcinoma. J Clin Invest..

[80] Wu T, Lin C, Chang C, Lin T, Martel J, Ko Y (2019). Gut commensal *Parabacteroides goldsteinii* plays a predominant role in the anti-obesity effects of polysaccharides isolated from *Hirsutella sinensis*. Gut..

[81] Wang K, Liao M, Zhou N, Bao L, Ma K, Zheng Z, Wang Y, Liu C, Wang W, Wang J, Liu SJ, Liu H (2019). *Parabacteroides distasonis* alleviates obesity and metabolic dysfunctions via production of succinate and secondary bile acids. Cell Rep..

[82] Benson AK, Kelly SA, Legge R, Ma F, Low SJ, Kim J, Zhang M, Oh PL, Nehrenberg D, Hua K, Kachman SD, Moriyama EN, Walter J, Peterson DA, Pomp D (2010). Individuality in gut microbiota composition is a complex polygenic trait shaped by multiple environmental and host genetic factors. Proc Natl Acad Sci U S A..

[83] Ross EM, Moate PJ, Marett LC, Cocks BG, Hayes BJ (2013). Metagenomic predictions: from microbiome to complex health and environmental phenotypes in humans and cattle. PLoS One..

[84] Xue MY, Sun HZ, Wu XH, Liu JX, Guan LL (2020). Multi-omics reveals that the rumen microbiome and its metabolome together with the host metabolome contribute to individualized dairy cow performance. Microbiome..

[85] Rosenberg E, Zilber-Rosenberg I (2018). The hologenome concept of evolution after 10 years. Microbiome..

[86] Nozawa K, Kawabata-Shoda E, Doihara H, Kojima R, Okada H, Mochizuki S, Sano Y, Inamura K, Matsushime H, Koizumi T, Yokoyama T, Ito H (2009). TRPA1 regulates gastrointestinal motility through serotonin release from enterochromaffin cells. Proc Natl Acad Sci U S A..

[87] Cho H, Callaghan B, Bron R, Bravo DM, Furness JB (2014). Identification of enteroendocrine cells that express TRPA1 channels in the mouse intestine. Cell Tissue Res..

[88] Bertin S, Aoki-Nonaka Y, Lee J, de Jong PR, Kim P, Han T, Yu T, To K, Takahashi N, Boland BS, Chang JT, Ho SB, Herdman S, Corr M, Franco A, Sharma S, Dong H, Akopian AN, Raz E (2017). The TRPA1 ion channel is expressed in CD4+ T cells and restrains T-cell-mediated colitis through inhibition of TRPV1. Gut..

[89] Jakobsson A, Westerberg R, Jacobsson A (2006). Fatty acid elongases in mammals: their regulation and roles in metabolism. Prog Lipid Res..

[90] Gregory MK, Geier MS, Gibson RA, James MJ (2013). Functional characterization of the chicken fatty acid elongases. J Nutr..

[91] Pauter AM, Olsson P, Asadi A, Herslöf B, Csikasz RI, Zadravec D, Jacobsson A (2014). Elovl2 ablation demonstrates that systemic DHA is endogenously produced and is essential for lipid homeostasis in mice. J Lipid Res..

[92] Yue F, Cheng Y, Breschi A, Vierstra J, Wu W, Ryba T (2014). A comparative encyclopedia of DNA elements in the mouse genome. Nature..

[93] Gregory MK, Gibson RA, Cook-Johnson RJ, Cleland LG, James MJ (2011). Elongase reactions as control points in long-chain polyunsaturated fatty acid synthesis. PLoS One..

[94] Jehl F, Desert C, Klopp C, Brenet M, Rau A, Leroux S (2019). Chicken adaptive response to low energy diet: main role of the hypothalamic lipid metabolism revealed by a phenotypic and multi-tissue transcriptomic approach. BMC Genomics..

[95] Yao C, Spurlock DM, Armentano LE, Page CD, VandeHaar MJ, Bickhart DM (2013). Random forests approach for identifying additive and epistatic single nucleotide polymorphisms associated with residual feed intake in dairy cattle. J Dairy Sci..

[96] Balamatsias D, Kong AM, Waters JE, Sriratana A, Gurung R, Bailey CG, Rasko JEJ, Tiganis T, Macaulay SL, Mitchell CA (2011). Identification of P-Rex1 as a novel Rac1-Guanine nucleotide exchange factor (GEF) that promotes actin remodeling and GLUT4 protein trafficking in adipocytes. J Biol Chem..

[97] Xue R, Lynes MD, Dreyfuss JM, Shamsi F, Schulz TJ, Zhang H, Huang TL, Townsend KL, Li Y, Takahashi H, Weiner LS, White AP, Lynes MS, Rubin LL, Goodyear LJ, Cypess AM, Tseng YH (2015). Clonal analyses and gene profiling identify genetic biomarkers of the thermogenic potential of human brown and white preadipocytes. Nat Med..

[98] Munyaka PM, Nandha NK, Kiarie E, Nyachoti CM, Khafipour E (2016). Impact of combined beta-glucanase and xylanase enzymes on growth performance, nutrients utilization and gut microbiota in broiler chickens fed corn or wheat-based diets. Poult Sci..

[99] Pourabedin M, Guan L, Zhao X (2015). Xylo-oligosaccharides and virginiamycin differentially modulate gut microbial composition in chickens. Microbiome..

[100] Weiss WP, Wyatt DJ, McKelvey TR (2008). Effect of feeding propionibacteria on milk production by early lactation dairy cows. J Dairy Sci..

[101] Litsanov B, Brocker M, Bott M (2012). Toward homosuccinate fermentation: metabolic engineering of *Corynebacterium glutamicum* for anaerobic production of succinate from glucose and formate. Appl Environ Microbiol..

[102] Stanley D, Denman SE, Hughes RJ, Geier MS, Crowley TM, Chen H, Haring VR, Moore RJ (2012). Intestinal microbiota associated with differential feed conversion efficiency in chickens. Appl Microbiol Biot..

[103] Sergeant MJ, Constantinidou C, Cogan TA, Bedford MR, Penn CW, Pallen MJ (2014). Extensive microbial and functional diversity within the chicken cecal microbiome. PLoS One..

[104] Annison EF, Hill KJ, Kenworthy R (1968). Volatile fatty acids in the digestive tract of the fowl. Brit J Nutr..

[105] Gasaway WC (1976). Seasonal variation in diet, volatile fatty acid production and size of the cecum of roch ptarmigan. Comp Biochem Physiol A Comp Physiol..

[106] Gasaway WC (1976). Volatile fatty acids and metabolizable energy derived from cecal fermentation in the willow ptarmigan. Comp Biochem Physiol A Comp Physiol..

[107] Pierre JF, Martinez KB, Ye H, Nadimpalli A, Morton TC, Yang J, Wang Q, Patno N, Chang EB, Yin DP (2016). Activation of bile acid signaling improves metabolic phenotypes in high-fat diet-induced obese mice. Am J Physiol Gastrointest Liver Physiol..

[108] Gao X, Xie Q, Kong P, Liu L, Sun S, Xiong B (2017). Polyphenol- and caffeine-rich postfermented pu-erh tea improves diet-induced metabolic syndrome by remodeling intestinal homeostasis in mice. Infect Immun..

[109] Liu J, Li Y, Yang P, Wan J, Chang Q, Wang TTY, Lu W, Zhang Y, Wang Q, Yu LL (2017). Gypenosides reduced the risk of overweight and insulin resistance in C57BL/6J mice through modulating adipose thermogenesis and gut microbiota. J Agr Food Chem..

[110] Depommier C, Van Hul M, Everard A, Delzenne NM, De Vos WM, Cani PD (2020). Pasteurized *Akkermansia muciniphila* increases whole-body energy expenditure and fecal energy excretion in diet-induced obese mice. Gut Microbes..

[111] Altaher YW, Jahromi MF, Ebrahim R, Zulkifli I, Liang JB (2015). *Lactobacillus Pentosus* Ita23 and *L. Acidipiscis* Ita44 enhance feed conversion efficiency and beneficial gut microbiota in broiler chickens. Braz J Poult Sci..

[112] Gao P, Ma C, Sun Z, Wang L, Huang S, Su X (2017). Feed-additive probiotics accelerate yet antibiotics delay intestinal microbiota maturation in broiler chicken. Microbiome..

