# Supplementary figures and images for: Joint contributions of the gut microbiota and host genetics to feed efficiency in chickens
Source: Microbiome. 2021 Jun 1;9:126. doi: 10.1186/s40168-021-01040-x (PMC8171024; doi:10.1186/s40168-021-01040-x)

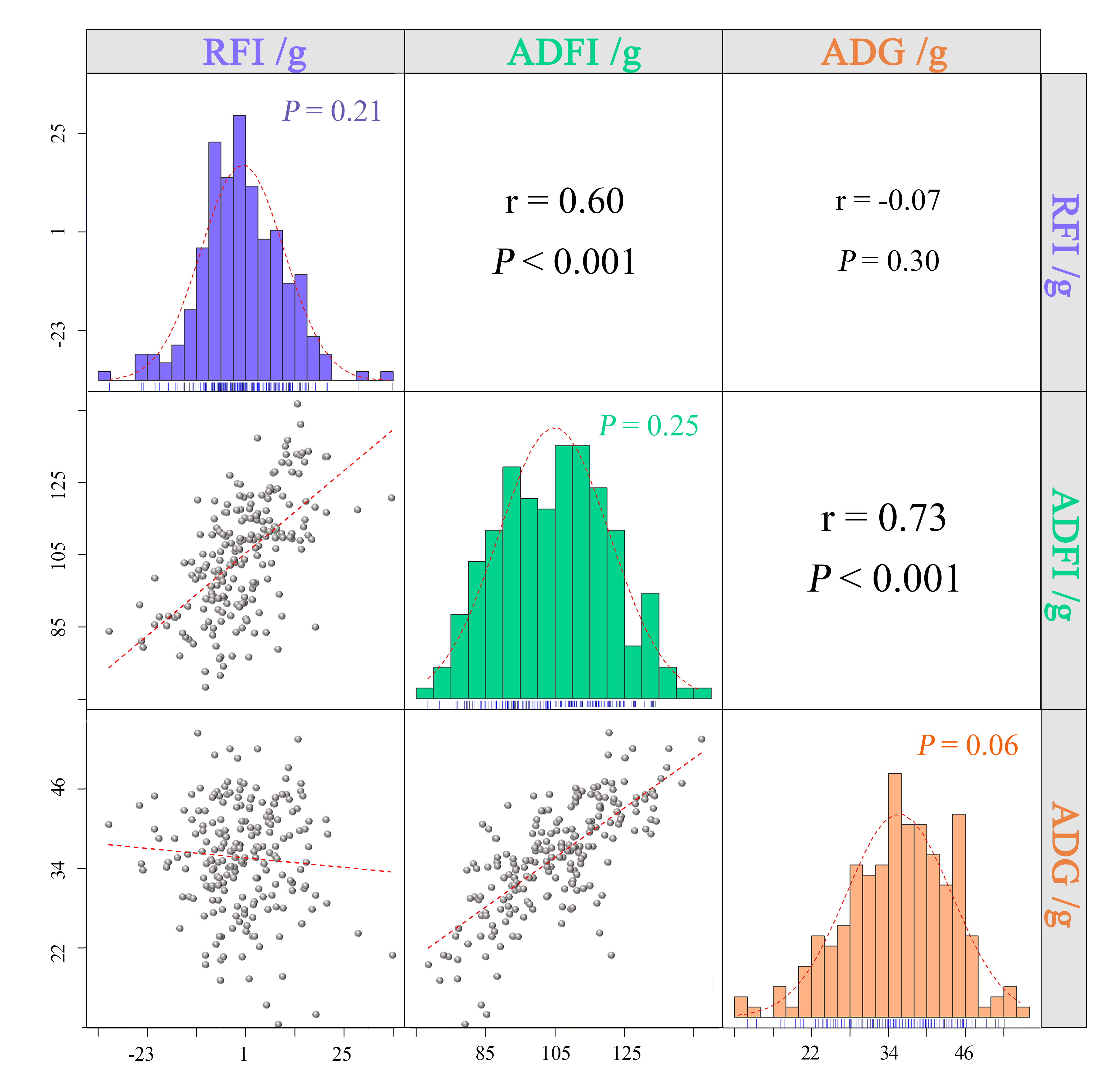

Supplement: Supplementary file 4 — Additional file 3: Figure S1. Distribution and correlation of host phenotypes. [file 40168_2021_1040_MOESM4_ESM.tif]

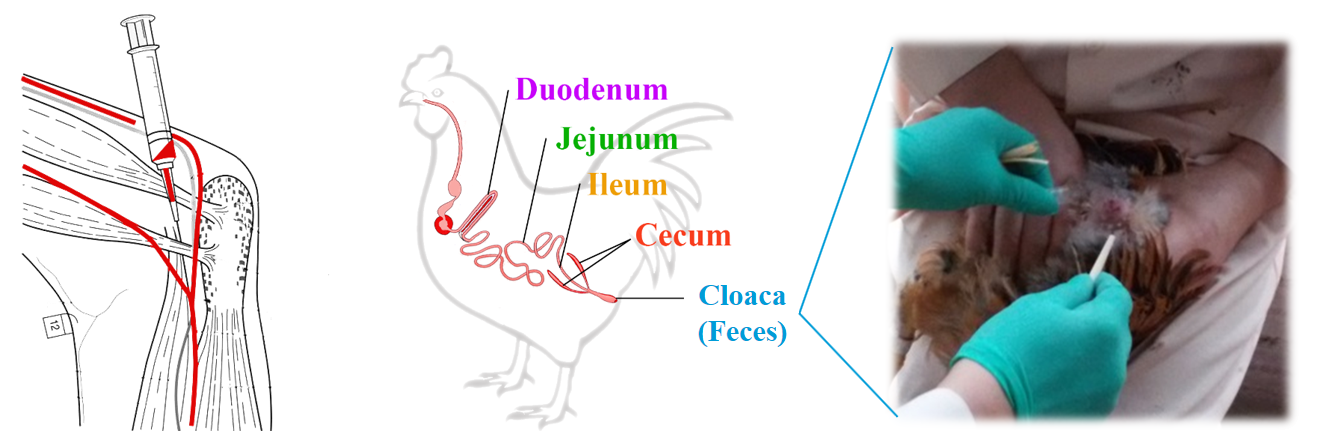

Supplement: Supplementary file 5 — Additional file 4: Figure S2. Collection of blood from the wing vein and the sampling sites of the gut content and feces. [file 40168_2021_1040_MOESM5_ESM.tif]

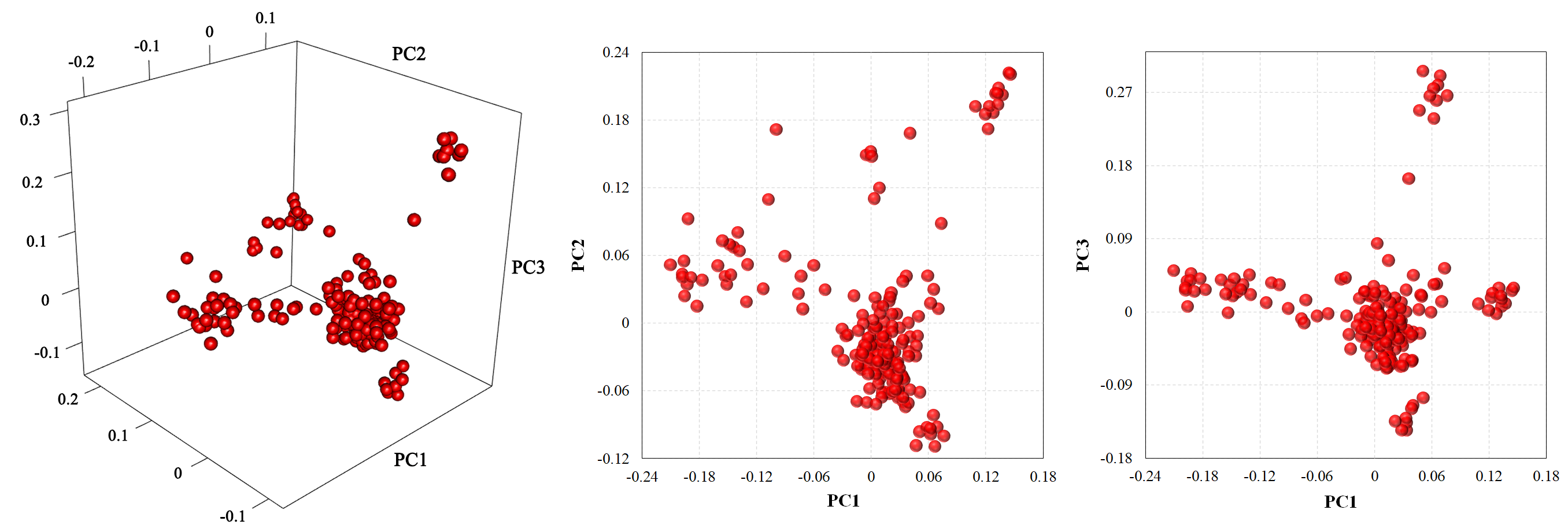

Supplement: Supplementary file 6 — Additional file 5: Figure S3. The principal component analysis of the host genetics. [file 40168_2021_1040_MOESM6_ESM.tif]

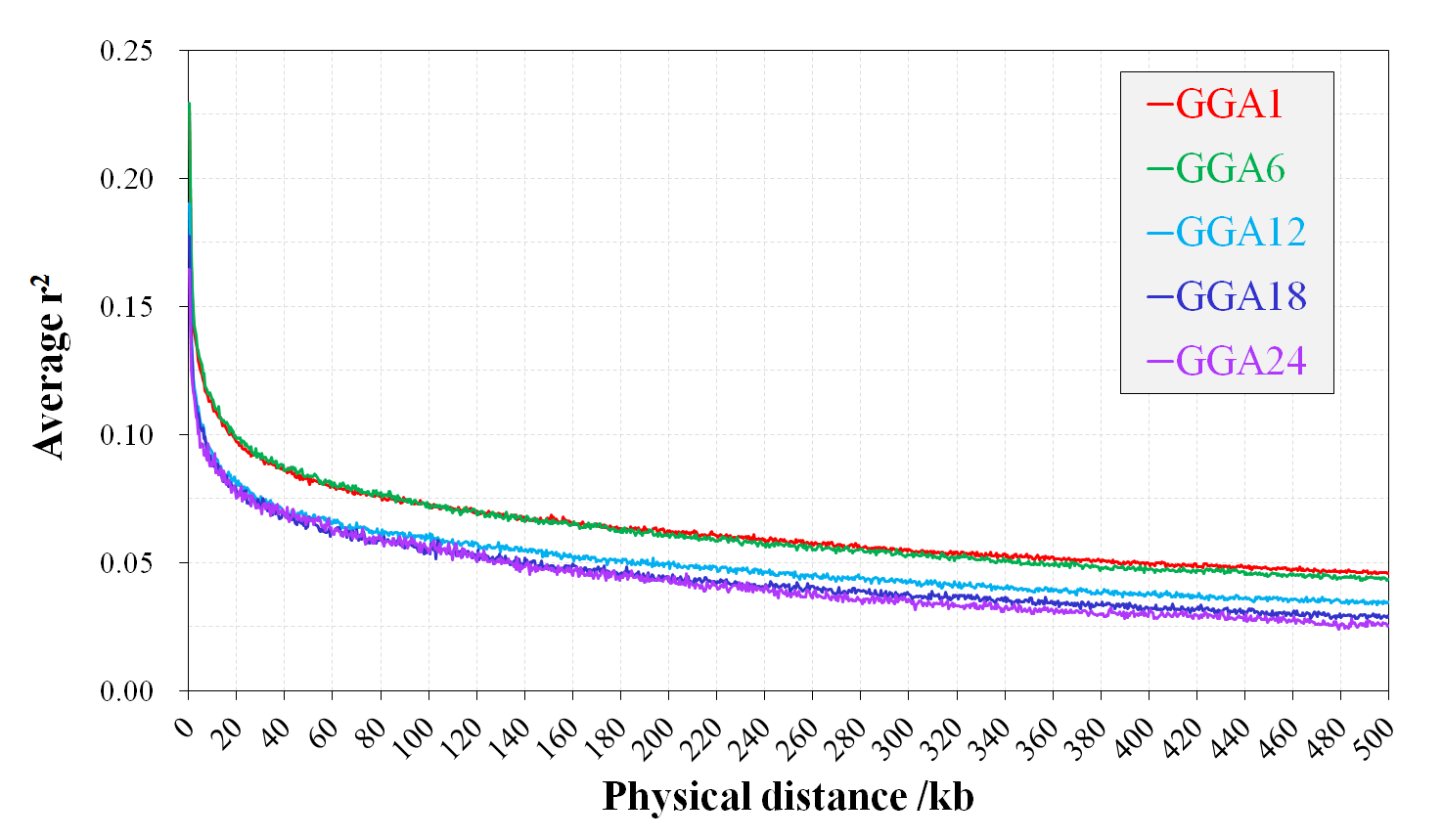

Supplement: Supplementary file 9 — Additional file 8: Figure S4. Decay of chromosome-wide linkage disequilibrium (LD). [file 40168_2021_1040_MOESM9_ESM.tif]

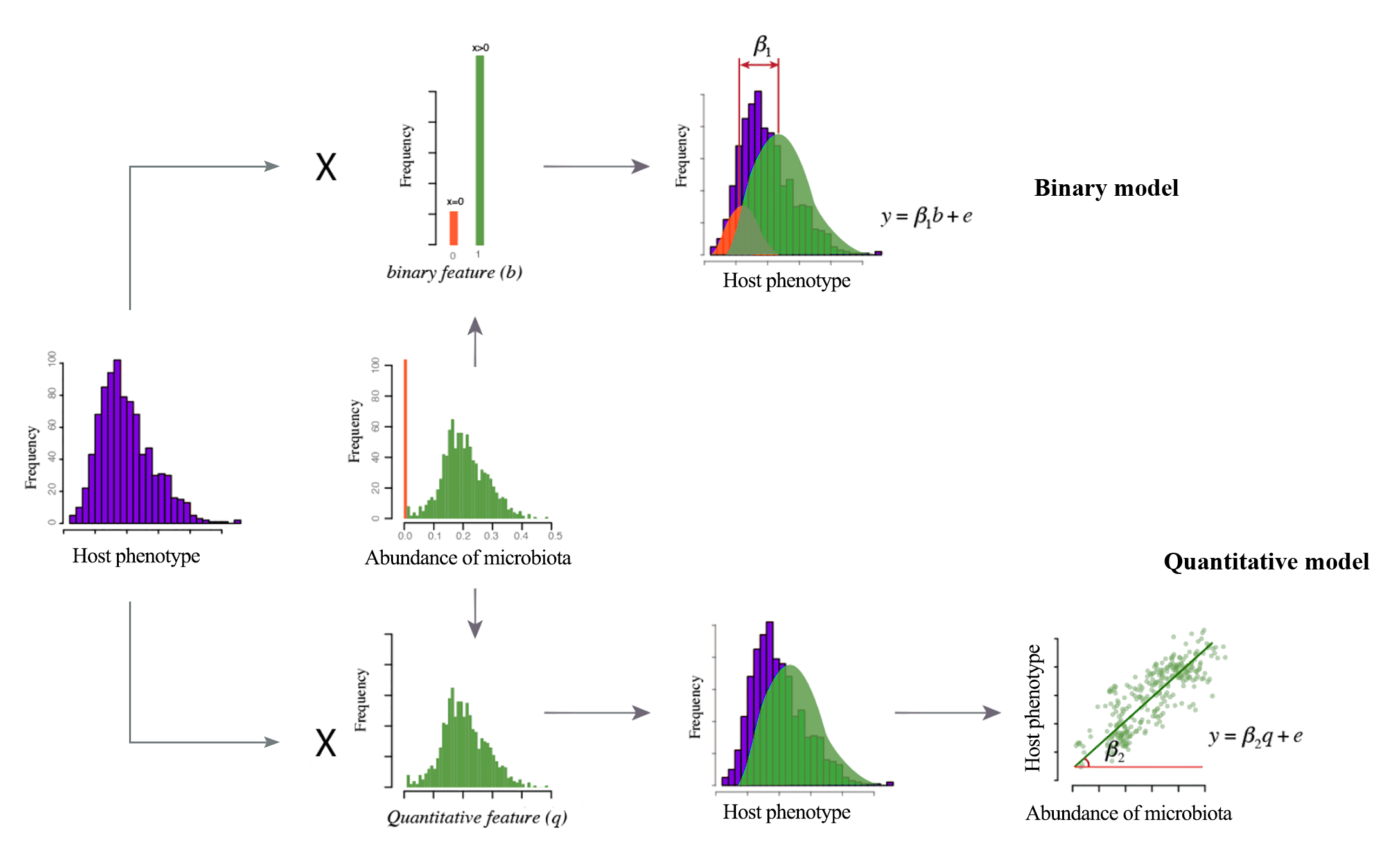

Supplement: Supplementary file 10 — Additional file 9: Figure S5. Workflow of the two-part model (cited from Fu et al. [53]). [file 40168_2021_1040_MOESM10_ESM.tif]

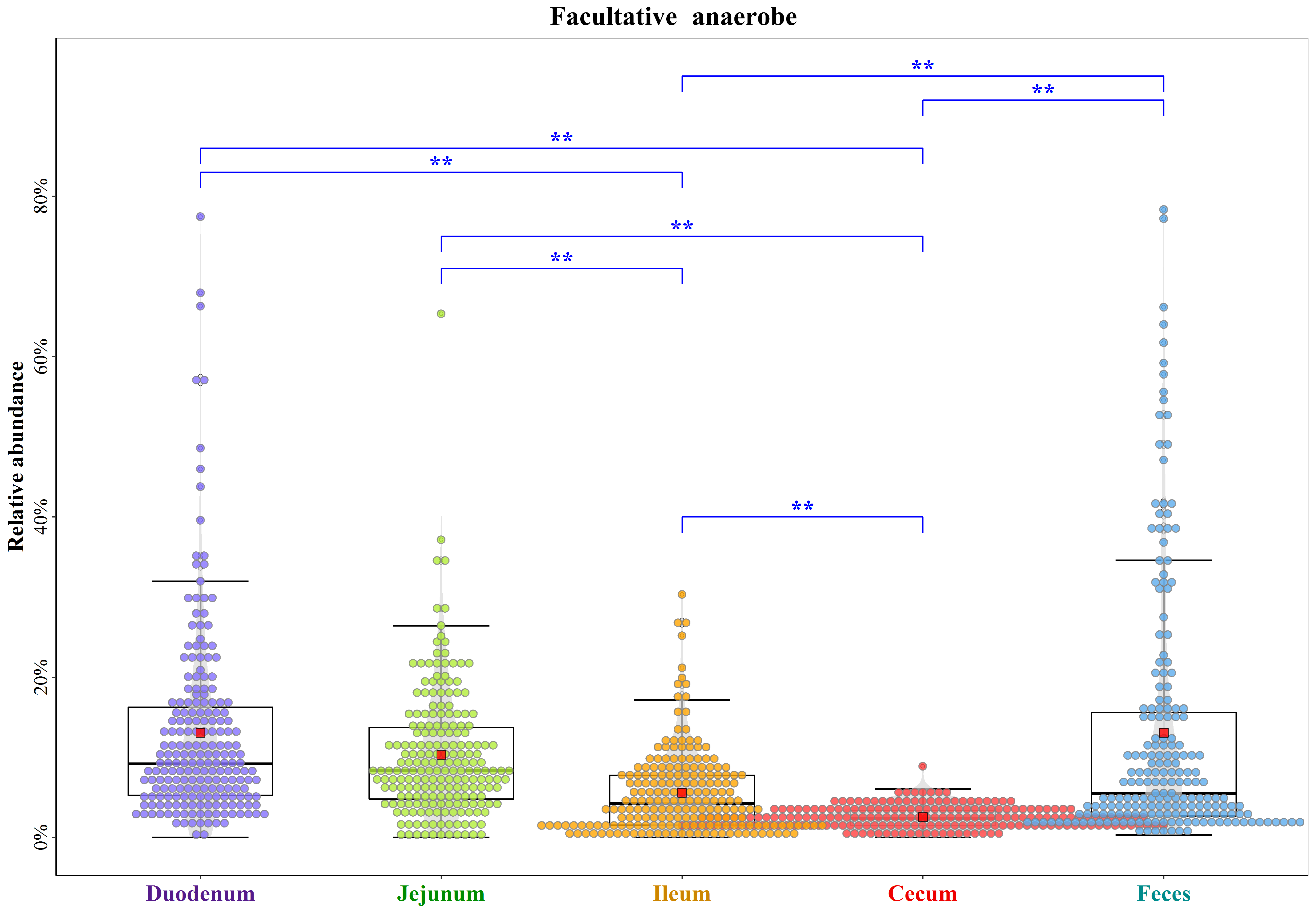

Supplement: Supplementary file 11 — Additional file 10: Figure S6. Differences in the relative abundance of facultative anaerobes within the four gut segments and feces. [file 40168_2021_1040_MOESM11_ESM.tif]

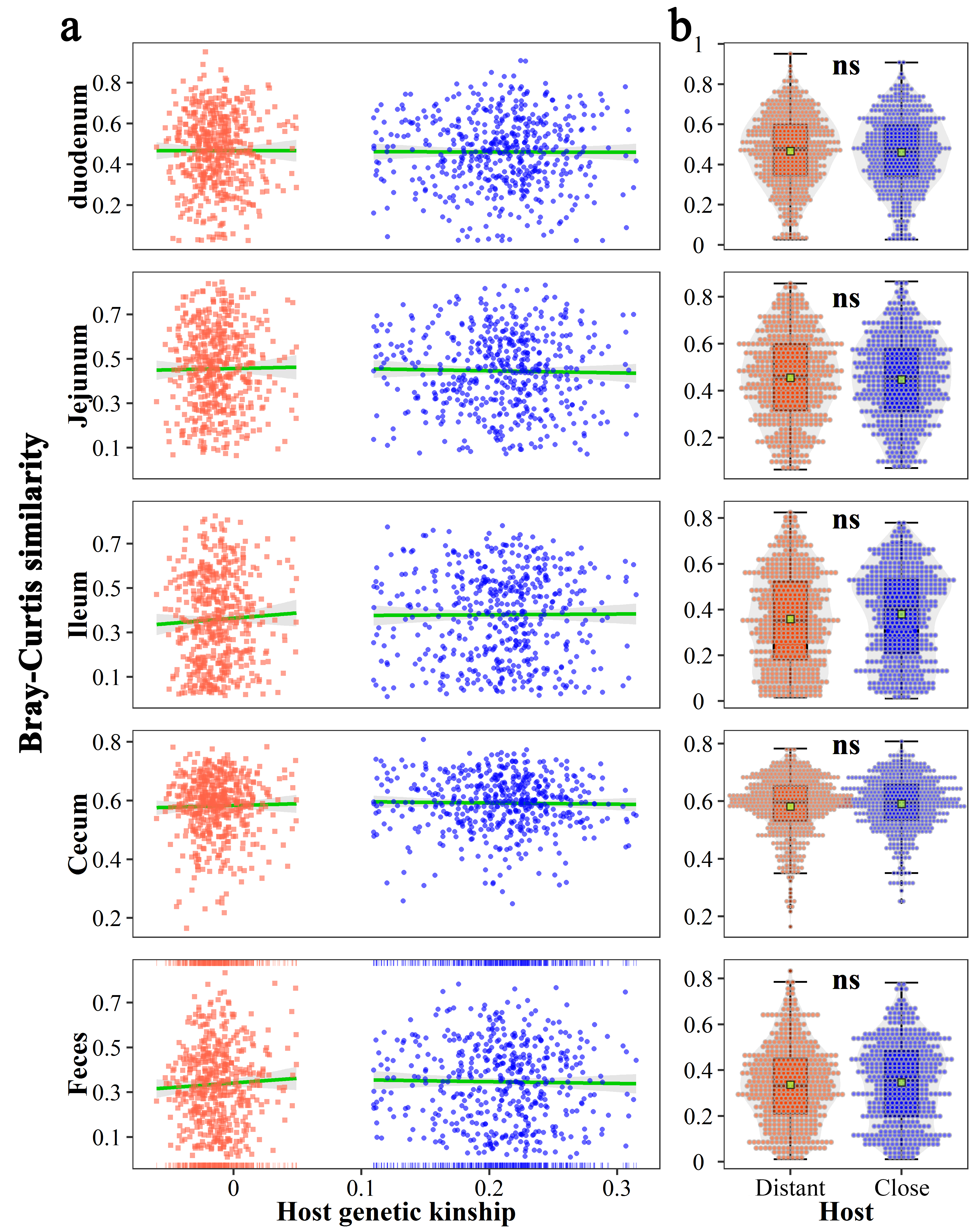

Supplement: Supplementary file 17 — Additional file 16: Figure S7. Comparison of the gut microbial similarity between genetically distinct relatives. (a) Correlations between host genetic kinship and the Bray-Curtis distance in the five sampling sites. (b) Comparison of the Bray-Curtis distance between more distant and close relatives. [file 40168_2021_1040_MOESM17_ESM.tif]

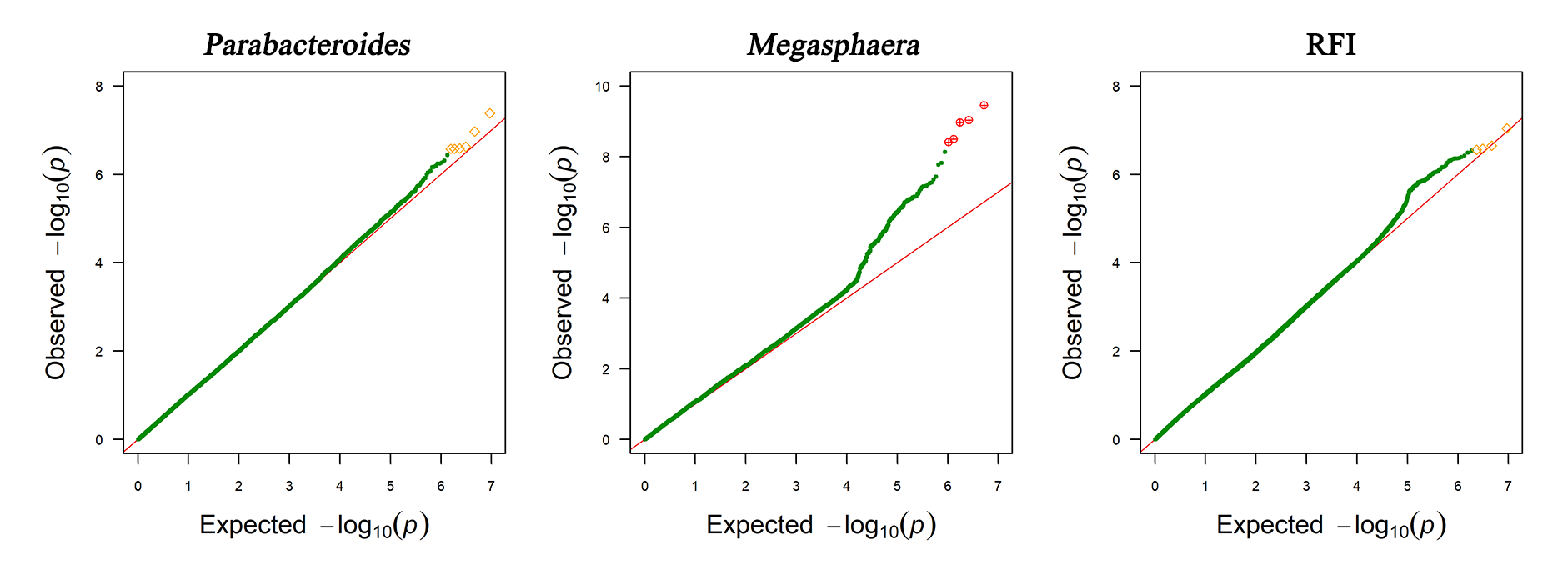

Supplement: Supplementary file 20 — Additional file 19: Figure S8. The corresponding Q-Q plots for the genome-wide associations studies. [file 40168_2021_1040_MOESM20_ESM.tif]
