# Supplementary material for: Joint contributions of the gut microbiota and host genetics to feed efficiency in chickens
Source: Microbiome. 2021 Jun 1;9:126. doi: 10.1186/s40168-021-01040-x (PMC8171024; doi:10.1186/s40168-021-01040-x)
Supplement: Supplementary file 8 — Additional file 7: Text S1. Sequence processes and quality control pipeline. [file 40168_2021_1040_MOESM8_ESM.docx]

# Supplementary methods

# Host sequence processes

A typical analysis pipeline for WGS data involves the removal of low-quality sequencing reads and the alignment of sequencing reads to a reference genome followed by variant calling. A post-alignment step to remove all but one duplicate is important for accurate variant calling. The resulting alignments were processed according to the best practices for the Genome Analysis Toolkit (**GATK**, ver 3.7) [1]. The pipelines used to process WGS data were follow:


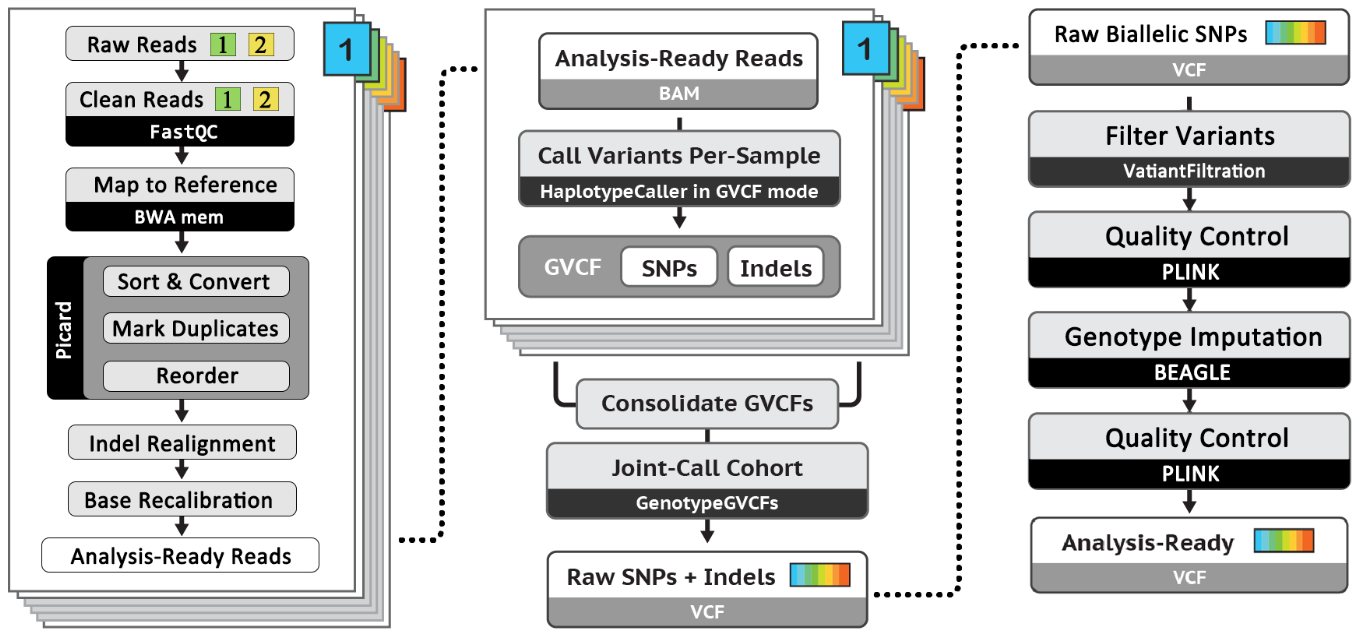


To avoid reads with artificial bias, quality control was conducted using FastQC (ver 11.7) with the following parameters: (1) reads that aligned to adaptors or primers with no more than two mismatches; (2) reads with more than 10% unknown bases; (3) reads with more than 30% of low-quality bases (quality value ≤20) in one read. The clean reads of the host were mapped on the reference genome (Galgal5) using BWA mem (ver 0.7.15) [2] with the default parameters. We further sorted the alignment reads and removed the duplicates using the Picard toolkit (ver 1.119). The resulting alignments were indexed using SAMtools (ver 1.6) [3]. Subsequently, GATK was used for SNP calling, following GATK best practices, in which realignment and recalibration were included.

SNP calling and genotyping were performed using the HaplotypeCaller module in GATK. To obtain high-quality SNPs, we set a minimum quality score of 20 for both base quality and mapping quality to call variants [4]. The average genome coverage was 95.25%, which allowed us to call variants with high coverage. The SNPs of each individual bird were combined to obtain a common set of SNP data, and the resulting dataset was subjected to filtering based on rigorous criteria using the GATK VariantFiltration module. The filter expressions were "QD < 10.0 || MQ < 40.0 || FS > 60.0 || MQRankSum < -12.5 || ReadPosRankSum < -8.0". Additionally, if more than 3 SNPs were clustered in one 10-bp window, all the SNPs were considered false positives and removed [4].

Only biallelic SNPs were used to achieve more robust quality control using PLINK (ver 1.9) [5] with the following parameters: sample call rate > 90%, SNP call rate > 95%, minor allele frequencies > 5% and Hardy–Weinberg equilibrium *P*-value < 10^−5^. The remaining SNPs and individuals were used for imputation in BEAGLE (ver 4.0) [6], and PLINK analysis was re-performed using the same above-described criteria. The final set included 9,335,193 SNPs (87.82% of the SNPs were found in the SNP database), which were used in downstream analysis.

# 16S rRNA sequence processes

Raw reads with exact matches to the barcodes were assigned to respective samples and identified as valid sequences. The low-quality reads that met the following criteria were filtered: (1) read lengths shorter than 150 bp; (2) reads containing ambiguous bases; (3) mononucleotide repeats more than 8 bp; (4) average quality score less than 20. The high-quality paired-end reads with an overlap > 10 bp and without any mismatch were assembled using FLASH [7] and were subsequently processed and clustered into OTUs at 97% sequence identity using an open-reference OTU picking protocol in QIIME (ver 1.8.0) [8]. Singletons were filtered from the dataset, and OTUs with an average relative abundance < 10^−6^ were removed from the analysis.

**References**

1. Mckenna A, Hanna M, Banks E, Sivachenko A, Cibulskis K, Kernytsky A, et al. The Genome Analysis Toolkit: A MapReduce framework for analyzing next-generation DNA sequencing data. Genome Res. 2010;20(9):1297-303.

2. Li H, Durbin R. Fast and accurate short read alignment with Burrows-Wheeler transform. Bioinformatics. 2009;25(14):1754-60.

3. Li H, Handsaker B, Wysoker A, Fennell T, Ruan J, Homer N, et al. The sequence alignment/map format and SAMtools. Bioinformatics. 2009;25(16):2078-9.

4. Li H, Ruan J, Durbin R. Mapping short DNA sequencing reads and calling variants using mapping quality scores. Genome Res. 2008;18(11):1851-8.

5. Purcell S, Neale B, Todd-Brown K, Thomas L, Ferreira MA, Bender D, et al. PLINK: a tool set for whole-genome association and population-based linkage analyses. Am J Hum Genet. 2007;81(3):559-75.

6. Browning SR, Browning BL. Rapid and accurate haplotype phasing and missing-data inference for whole-genome association studies by use of localized haplotype clustering. Am J Hum Genet. 2007;81(5):1084-97.

7. Magoc T, Salzberg SL. FLASH: fast length adjustment of short reads to improve genome assemblies. Bioinformatics. 2011;27(21):2957-63.

8. Caporaso JG, Kuczynski J, Stombaugh J, Bittinger K, Bushman FD, Costello EK, et al. QIIME allows analysis of high-throughput community sequencing data. Nat Methods. 2010;7(5):335-6.
